# Evolution of intermolecular contacts with temperature and pressure in bromoethane and iodo­ethane – a comparative study

**DOI:** 10.1107/S2052520622010149

**Published:** 2022-11-09

**Authors:** Maciej Bujak, Anna Olejniczak, Marcin Podsiadło

**Affiliations:** a Faculty of Chemistry, University of Opole, Oleska 48, Opole, 45-052, Poland; b Faculty of Chemistry, Adam Mickiewicz University, Uniwersytetu Poznańskiego 8, Poznań, 61-614, Poland; CSIR–National Chemical Laboratory, India

**Keywords:** *in situ* crystallization, low temperature, high pressure, halogenated alkanes

## Abstract

At ambient conditions two liquids, bromo­ethane, C_2_H_5_Br (**MBE**, m.p. 154.6 K) and iodo­ethane, C_2_H_5_I (**MIE**, m.p. 162.1 K), have been crystallized under both isobaric and isochoric conditions using *in situ* low-temperature and high-pressure techniques. The single-crystal X-ray diffraction studies, supported by the isothermal compressibility and isobaric differential scanning calorimetry measurements, allowed the monitoring and analysis at low temperature and high pressure of the effects of externally induced changes in **MBE** and **MIE**. The monoclinic **MBE** and **MIE** crystals are found to be isostructural. They are stable, in the investigated regimes, *i.e.* down to 100 K/0.1 MPa and up to ∼295 K/3.7 GPa, without undergoing any symmetry or phase change. Both the contraction and compression is mainly noted for the intermolecular separation distances. The Hirshfeld surface analysis of intermolecular contacts clearly shows the nature of occurrence and hierarchy of interactions pointing out the role played by Br/I⋯Br/I contacts, in particular at ambient temperature and high-pressure conditions. The preferences for the formation of intermolecular contacts in **MIE** are more pronounced and do not require such extreme conditions as in the case of **MBE**. It is notable that the Br/I⋯Br/I and Br/I⋯H contacts could be basically classified as type-I interactions.

## Abstract 2

Preferences for formation and competition between halogen⋯halogen and halogen⋯H contacts have been studied for model compounds of monohalogenated ethanes.

## 1. Introduction

The *in situ* low-temperature and high-pressure experimental techniques of structural chemistry are successfully used in investigations of compounds that are liquids or gases at ambient conditions. Both these techniques have been relatively easily implemented into X-ray diffraction studies to investigate the limited factors that are usually not available at ambient conditions, including weak intermolecular interactions, polymorphism and structural transformations (*e.g.* Brodalla *et al.*, 1985 ▸; Goeta & Howard, 2004 ▸; Kirchner *et al.*, 2010 ▸; Bujak & Katrusiak, 2010 ▸; Boese, 2014 ▸; Maloney *et al.*, 2014 ▸; Li *et al.*, 2016 ▸; Dey *et al.*, 2019 ▸; Boldyreva, 2019 ▸; Katrusiak, 2019 ▸). The first issue, associated with intermolecular interactions, relates to both structural transformations of materials and the products of those changes – different phases and polymorphic forms that could be induced by applying external temperature or pressure. It is also worth noting that variations in those physicochemical crystallization parameters effectively affect the structure and properties of the solid-state products that are obtained. These apply to crystallization of new compounds as well as new forms of known compounds including their cocrystals. The X-ray diffraction studies, besides the changes of geometrical parameters, can also indicate the importance and energy of the specific interactions responsible for the unique behaviour of a given crystalline material.

The relatively small, and preferably showing well defined types of interactions, alkanes and their derivatives could be considered as the model compounds for the studies of molecular materials at non-ambient conditions (*e.g.* Boese *et al.*, 1999 ▸; Dziubek *et al.*, 2009 ▸; Bujak *et al.*, 2019 ▸). The previous investigations on the whole group of chloro­ethanes clearly showed the differences within their isomers arising from the different nature of intermolecular interactions. 1,2-Di­chloro­ethane (12DCE), in contrast to its asymmetrically substituted isomeric 1,1-di­chloro­ethane (11DCE) shows Cl⋯Cl contacts within a regime of the sum of the van der Waals radii (Boese *et al.*, 1992 ▸; Bujak *et al.*, 2004 ▸, 2008*a* ▸; Bondi, 1964 ▸). Similar behaviour was found for tri­chloro­ethanes, *i.e.* 1,1,2- and the more asymmetrically substituted 1,1,1-tri­chloro­ethane (Bujak *et al.*, 2008*b* ▸; Bujak *et al.*, 2011 ▸). Therefore both 11DCE and 111TCE could be considered as ‘crystalline gases’ with no intermolecular interactions shorter than the sums of the van der Waals radii of respective atoms. In these crystals, the lack of interactions between asymmetrically substituted ethane molecules could be explained in terms of the specific shape of molecules that, in turn, is related to both the mismatch of electrostatic potential on the surfaces of molecules and also steric hindrances.

The simplest among chloro­ethanes (mono)chloro­ethane (C_2_H_5_Cl, **MCE**) has been found to crystallize in two different phases depending on the crystallization conditions (Podsiadło *et al.*, 2012 ▸). The low-temperature isobaric freezing resulted in the monoclinic *P*2_1_/*n* phase I, whereas the high-pressure crystallization, at isochoric conditions, yielded the hexagonal *P*6_3_/*m* phase II. Besides the differences associated with crystal symmetry and structure, the main dissimilarity between these two phases relates to intermolecular interactions. The low-temperature monoclinic phase I, similar to aforementioned 11DCE and 111TCE, shows no intermolecular contacts within the sums of the van der Waals radii regime, whereas in the high-pressure hexagonal phase II the chloro­ethane molecules are joined together by all possibly expected Cl⋯Cl, Cl⋯H and H⋯H interactions.

Herein, continuing our studies on simple compounds under extreme conditions, we present our structural investigations on two analogous halogeno­ethanes: (mono)bromo­ethane, C_2_H_5_Br (**MBE**) and (mono)iodo­ethane, C_2_H_5_I, (**MIE**). Similar to **MCE**, **MBE** and **MIE** are liquids under ambient conditions (Lide, 2007 ▸). Both **MBE** and **MIE** have been *in situ* crystallized at low-temperature and high-pressure conditions, and subsequently their single-crystal structures have been determined at various low-temperature ambient-pressure and ambient-temperature high-pressure points. The X-ray diffraction studies have been supported by ambient-temperature compressibility and thermoanalytical ambient-pressure differential scanning calorimetry measurements. To further understand and compare the processes occurring with halogen atom exchange as well as the influence of decreasing temperature and increasing pressure on the formation and nature of interactions, in these two simple alkane derivatives, Hirshfeld surface analysis of intermolecular contacts has been applied.

## 2. Experimental

Bromo­ethane, C_2_H_5_Br (**MBE**) and iodo­ethane, C_2_H_5_I (**MIE**) (both ReagentPlus, 99%, Sigma-Aldrich) were used directly, as commercially supplied, in the single-crystal X-ray diffraction, compressibility and differential scanning calorimetry experiments.

### 2.1. *In situ* low-temperature crystallization

The colourless liquid samples of **MBE** and **MIE** were sealed in thin-walled glass capillaries (internal diameter of 0.3 mm and wall of 0.01 mm thickness) and mounted on a diffractometer. The temperature was controlled by an Oxford Cryosystems Cryostream cooler. The samples initially froze as the polycrystalline materials. Then, the reduction of the number of crystal seeds, by cycling the temperature close to melting points of those compounds (Lide, 2007 ▸) followed by slow temperature decrease, allowed for the growth of sufficiently large single crystals used for the collection of X-ray diffraction data.

### 2.2. *In situ* high-pressure crystallization

The high-pressure experiments of **MBE** and **MIE** were performed using a modified Merrill–Bassett diamond-anvil cell (DAC) (Merrill & Bassett, 1974 ▸; Bassett, 2009 ▸). The diameter of the diamond culets was 0.8 mm. The gasket was made of 0.1 mm (**MBE**) and 0.3 mm (**MIE**) thick steel foil with a spark-eroded hole of 0.4 mm in diameter for both **MBE** and **MIE** (Katrusiak, 1999 ▸). The ruby-fluorescence method, using a BETSA PRL spectrometer, was utilized to measure the pressure in the DAC (Barnett *et al.*, 1973 ▸; Piermarini *et al.*, 1975 ▸) with the accuracy of ∼0.02 GPa.

**Figure 1:**
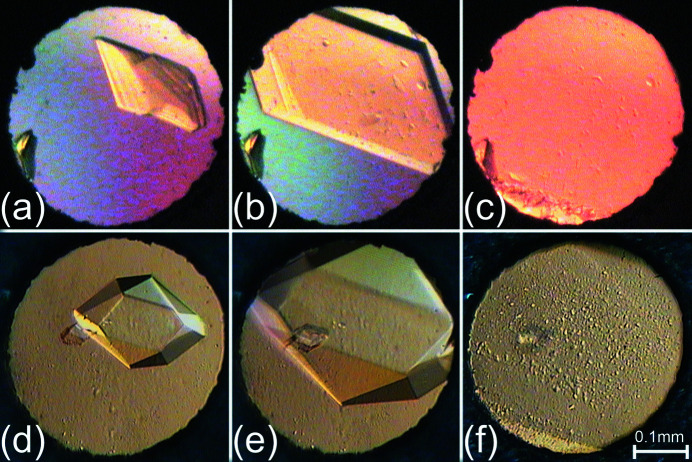
Stages of isochoric, with decreasing temperature, growth of the **MBE** (*a*)–(*c*) and **MIE** (*d*)–(*f*) single crystals in a diamond-anvil cell (*cf.* Figs. S1 and S2).

Several attempts to crystallize **MBE** and **MIE** were undertaken. Eventually, the best results were obtained for initial squeezing of the liquid samples, up to ∼2 GPa in the DAC, followed by quenching the DAC (with a non-solidified sample) in liquid nitro­gen. This resulted in the polycrystalline **MBE** and **MIE** samples being obtained. Then the DAC, with the polycrystalline sample, was allowed to reach room temperature. The single crystals of both **MBE** and **MIE** were obtained under isochoric conditions: the DAC with the polycrystalline material was heated using a hot-air gun until all but one crystal seed were melted. In the next step the single crystal grew, as the DAC was cooled slowly to ambient temperature in a controlled manner, and eventually the sample filled the whole volume of the high-pressure chamber. The progress in growing of the single-crystal samples, at selected high-pressure points, of **MBE** and **MIE** is shown in Figs. 1 ▸, S1 and S2 (in the supporting information). For all high-pressure experiments, except of those for **MBE** at 3.07 (2) and 3.87 (2) GPa (in which the single-crystal sample previously obtained at 2.13 (2) GPa was slowly pressurized), after the data collection processes, the pressure in the DAC was increased and the crystals were melted. Then the ‘new’ single-crystal samples were isochorically grown again at higher pressure.

### 2.3. Data collection, data reduction, structure solution and refinement

The various-temperature ambient-pressure (0.1 MPa) and room-temperature [295 (2) K] high-pressure diffraction data were collected on a KUMA KM4-CCD diffractometer with an Eos detector (variable temperature for **MBE,** and variable pressure for **MBE** and **MIE**) and on an Xcalibur Eos diffractometer (variable temperatures for **MIE**), both with the graphite-monochromated Mo *Kα* radiation.

The first low-temperature datasets, for both **MBE** and **MIE**, were gathered at the same highest possible temperature, limited by the stability of the single-crystal samples, *i.e.* at 140.0 (1) K. Then the temperature of the crystal samples was slowly decreased and the diffraction data were collected at 120.0 (1) and 100.0 (1) K. For both **MBE** and **MIE** the reflections were measured using the ω-scan technique with *Δω* = 1.0° and *Δt =* 5 s exposure time.

The single-crystal samples of both **MBE** and **MIE**, pressurized in a DAC, were centred on a diffractometer using the shadow method (Budzianowski & Katrusiak, 2004 ▸). The first datasets were collected at a very similar pressure value ensuring the stability of the crystal samples during data collection processes, *i.e.* 1.83 (2) and 1.88 (2) GPa for **MBE** and **MIE**, respectively. Then the pressure was increased in three, on average, ∼0.6 GPa steps finally reaching 3.87 (2) and 3.62 (2) GPa for **MBE** and **MIE**, respectively. The room-temperature high-pressure intensity data were collected using the ω- and φ-scan techniques with Δω/Δφ = 0.75°. The exposure time was 25 and 35 s for **MBE** and **MIE**, respectively.

The *CrysAlisPro* program was used for the data collection, unit-cell refinement and data reduction (Rigaku Oxford Diffraction, 2015 ▸, 2020 ▸). All data were corrected for Lorentz, polarization and absorption effects (Rigaku Oxford Diffraction, 2020 ▸). The structures were solved by direct methods and refined with *SHELX* (Sheldrick, 2008 ▸, 2015 ▸). All C, Br and I atoms were refined with anisotropic displacement parameters. The comparison of the average ADP values for non-H atoms of **MBE** and **MIE** as well as unit-volume changes with temperature and pressure are depicted in Fig. S3. H-atom positions in all structures were located in difference Fourier maps and then a riding model was applied. The isotropic displacement parameters of the H atoms were fixed to 1.2 and 1.5*U*_eq_ of their carrier C atoms.

The asymmetric units and the labelling of C, Br and I atoms for both **MBE** and **MIE**, at all studied low-temperature and high-pressure points, were chosen in the same manner to show the structural relationship between the positions of corresponding atoms and intermolecular contacts as well as to the previously investigated monoclinic phase I of **MCE** (Podsiadło *et al.*, 2012 ▸).

The crystal data and structure determination summary for **MBE** and **MIE** at all low-temperature and high-pressure conditions are listed in Tables 1 ▸, 2 ▸ and S1–S4 (in the supporting information). The coefficients of thermal expansion and compressibility along with the Birch–Murnaghan coefficients, calculated using *PASCal* (Cliffe & Goodwin, 2012 ▸), are given in Tables S5–S8. The bond lengths, bond angles and the geometries of shortest intermolecular contacts are presented in Tables 3 ▸, 4 ▸ and S9–S14. Intermolecular contacts were compared using Hirshfeld surface analysis with *CrystalExplorer17* (Turner *et al.*, 2017 ▸; Spackman *et al.*, 2021 ▸). The structures were drawn using *Mercury* (Macrae *et al.*, 2020 ▸).

### 2.4. Differential scanning calorimetry measurements

The ambient-pressure differential scanning calorimetry (DSC) analyses were performed using a DSC8500 (Perkin Elmer) calorimeter with cooling and heating runs of 10 K min^−1^. Nitro­gen (20 ml min^−1^) was used as a purge gas during the experiments. The measurements were performed in the temperature range 112–298 K only finding characteristic heat anomalies associated with freezing/melting of **MBE** and **MIE**. The determined onset temperatures (heating runs) for those phase changes are 153.9 K and 162.9 K for **MBE** and **MIE**, respectively (Figs. S4 and S5).

### 2.5. Compressibility measurements

The room-temperature [295 (2) K] compressibility measurements between ambient pressure and ∼1.1 GPa were performed in the piston-and-cylinder apparatus (Baranowski & Moroz, 1982 ▸; Dziubek & Katrusiak, 2014 ▸). The pressure was increased in ∼20–30 MPa steps. The diagrams show no anomalies. The compressibility of both liquids is comparable, however, the larger changes in molecular volume for **MIE** (30.6 Å^3^) than for **MBE** (28.9 Å^3^) should be noted (Figs. S6 and S7).

## 3. Results and discussion

The low-temperature and high-pressure single-crystal X-ray diffraction data, for both **MBE** and **MIE**, in contrast to **MCE** (Podsiadło *et al.*, 2012 ▸), have revealed only one phase, without any indication of a phase transition or symmetry change from the collected diffraction patterns, in the studied temperature, *i.e.* down to 100 K/0.1 MPa and pressure *i.e.* up to ∼295 K/3.7 GPa range. This observation is further confirmed by the low-temperature ambient-pressure differential scanning calorimetry and ambient-temperature high-pressure compressibility measurements showing no anomaly upon decreasing temperature to 112 K/0.1 MPa and compression up to 295 K/1.1 GPa (Figs. S4–S7).

**Figure 2:**
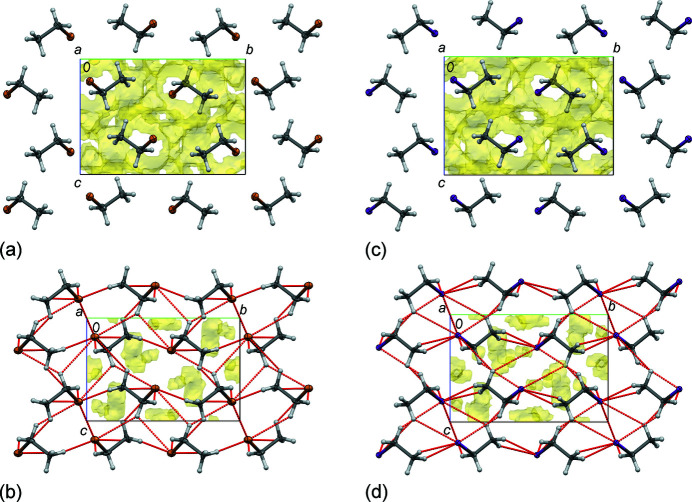
Structures of **MBE** and **MIE** at selected low-temperature and high-pressure conditions. **MBE** at (*a*) 140 K/0.1 MPa and (*b*) 295 K/3.87 GPa, **MIE** at (*c*) 140 K/0.1 MPa and (*d*) 295 K/3.62 GPa. The intermolecular space accessible to a probe with a radius of 0.3 Å and grid spacing of 0.2 Å is indicated in yellow. The void volume is (*a*) 26.5% (100.30 Å^3^), (*b*) 5.7% (16.48 Å^3^), (*c*) 28.5% (120.40 Å^3^) and (*d*) 6.9% (23.02 Å^3^), respectively. The red dotted lines indicate the intermolecular *X*⋯*X* and *X*⋯H contacts shorter than the sums of the van der Waals radii of respective atoms (*X* = Br for **MBE** and *X* = I for **MIE**, see Fig. 3 ▸). Displacement ellipsoids are plotted at the 25% probability level.

Furthermore, **MBE** and **MIE** crystals have been found to be isostructural. The single crystals of both compounds crystallize in monoclinic space group *P*2_1_/*n*, characterized by the very similar unit-cell parameters along with the positions of corresponding atoms and crystal packing arrangements (Tables 1 ▸, 2 ▸ and S1–S4, and Fig. 2 ▸).

We have also found that both **MBE** and **MIE** behave, in general, in a similar way with decreasing temperature and increasing pressure. All unit-cell parameters decrease, with the exception of the β angle that slightly increases, upon decreasing temperature. The largest linear contraction, of ∼1%, was noted for the *c* parameters. Overall the unit-cell volumes contract by ∼2% with decreasing temperature from 140 to 100 K. The expected larger changes were found at high pressure. Here, the unit-cell parameters compress, except for the slightly increased β angle in **MBE**, with increasing pressure from ∼1.9 to ∼3.7 GPa, by ∼4% and ∼10% for the most compressed *c* parameters and the unit-cell volumes, respectively (Tables 1 ▸, 2 ▸ and S1–S4). These results are consistent with the calculated coefficients of thermal expansion and compressibility (Tables S5–S8; Cliffe & Goodwin, 2012 ▸).

The asymmetric unit of both **MBE** and **MIE** contains one crystallographically unique ordered C_2_H_5_ *X* molecule (*X* = Br for **MBE** and *X* = I for **MIE**) that adopts a staggered conformation. There are no significant changes in intramolecular bond lengths and angles upon decreasing temperature and increasing pressure – all differences in molecular dimensions are within two e.s.d.s (Tables S9 and S10). As mentioned above, the principal molecular arrangements of structural components in both crystals are similar. However, the patterns of voids, as well as the systems of their intermolecular contacts, show some differences associated with the type of halogen atom and reflecting the hierarchy, number and type of contacts that are present at the specific thermodynamic conditions (Saraswatula & Saha, 2014 ▸; Saha *et al.*, 2018 ▸). A somewhat greater volume of voids for **MIE** than for **MBE** should be noted. Both the crystal contraction at low temperature and compression upon increasing pressure lead to a decrease of the free space in the crystal structures and the lengths of intermolecular contacts (Fig. 2 ▸).

**Figure 3:**
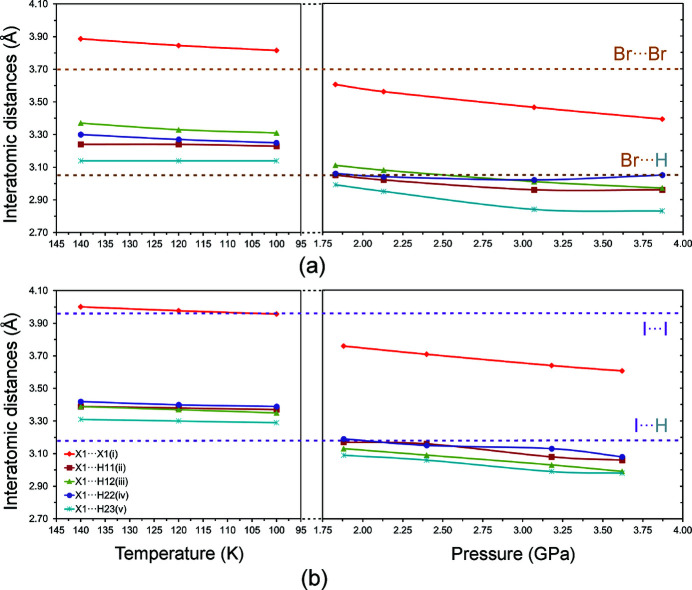
Evolution of the selected shortest intermolecular *X*⋯*X* and *X*⋯H distances (*X* = Br for **MBE**, and *X* = I for **MIE**) with decreasing temperature and increasing pressure for **MBE** (*a*, see Table 3 ▸) and **MIE** (*b*, see Table 4 ▸). The solid lines are for guiding the eye only. The dotted horizontal lines mark the sums of the van der Waals radii of respective atoms.

**MBE** and **MIE** crystals resemble, in the context of formation and the nature of interactions, the loose-packed crystals of unsymmetrically substituted chloro­ethanes, including **MCE** and also hexa­chloro­ethane (Bujak *et al.*, 2008*a* ▸, 2011 ▸, 2018 ▸; Podsiadło *et al.*, 2012 ▸). In crystalline **MBE** and **MIE** the molecules form *X*⋯*X* contacts, which on the basis of their geometrical parameters could be classified as type-I interactions. Considering geometrical criteria for the sums of the van der Waals radii there are no Br⋯Br contacts in any of the low-temperature determined structures of **MBE**. The first Br⋯Br contacts of 3.6040 (9) Å are noted in the structure determined at 295 K/1.83 GPa. The different behaviour is shown by **MIE** – lowering temperature at ambient pressure is sufficient to bring the iodo­ethane molecules to a I⋯I separation distance of 3.9564 (3) Å, at 100 K/0.1 MPa, which is equal to the distance determined for the van der Waals radii regime (3.96 Å, Bondi, 1964 ▸). The shortest distances between interacting *X* atoms have been found at the highest investigated pressure of ∼3.7 GPa. At this pressure both the Br⋯Br and I⋯I contacts are characterized by the separation distances that are, on average, ∼91% of the sums of the van der Waals radii of the respective atoms. To observe *X*⋯H contacts extreme conditions are needed. The first interactions of this type are observed in the high-pressure structures determined at 295 K/1.83 GPa and 295 K/1.88 GPa for **MBE** and **MIE**, respectively. The distances of interacting *X* and H atoms become closer, upon increasing pressure, and at the highest pressures of ∼3.7 GPa their shortest lengths of 2.83 Å and 2.98 Å for **MBE** and **MIE**, respectively, are on average ∼93% of the sums of the van der Waals radii of the respective atoms (Fig. 3 ▸, Tables 3 ▸, 4 ▸, S11 and S12; Bondi, 1964 ▸).

The H⋯H interatomic distances, relative to the van der Waals radii, are the longest. The extrapolation of the intermolecular distances up to ∼3.7 GPa shows that most of the shortest H⋯H distances become equal to the sum of the van der Waals radii at ∼3.2 GPa for **MBE**, whereas in the case of **MIE** even at the highest pressure of 3.62 GPa, all contacts are somewhat longer than the van der Waals radii regime.

**Figure 4:**
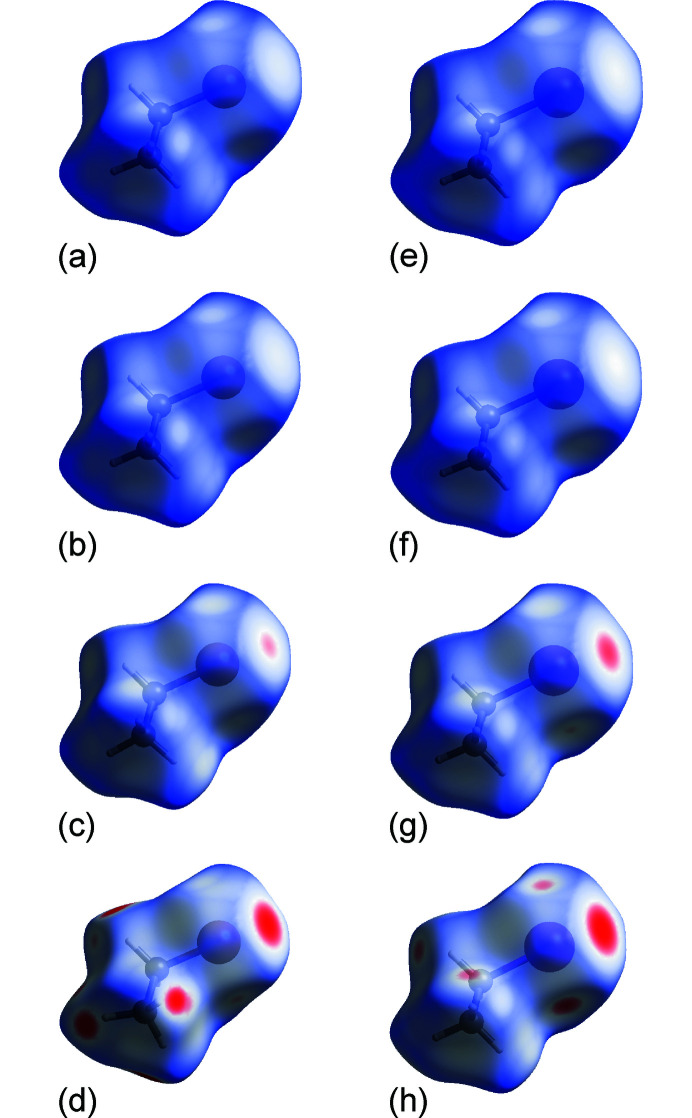
Hirshfeld surfaces mapped with *d*_norm_ (from −0.2000 to 1.2000) of **MBE** and **MIE** molecules at selected low-temperature and high-pressure conditions. **MBE** at (*a*) 140 K/0.1 MPa, (*b*) 100 K/0.1 MPa, (*c*) 295 K/1.83 GPa and (*d*) 295 K/3.87 GPa. **MIE** at (*e*) 140 K/0.1 MPa, (*f*) 100 K/0.1 MPa, (*g*) 295 K/1.88 GPa and (*h*) 295 K/3.62 GPa. The white, red and blue colours indicate contacts that are equal, shorter and longer, respectively, than the sums of the van der Waals radii of respective atoms.

Hirshfeld surface analysis, *i.e.* Hirshfeld surfaces along with the corresponding fingerprint plots, has been applied to further understand, visualize and separate contributions of different types of intermolecular contacts in **MBE** and **MIE** (Turner *et al.*, 2017 ▸; Spackman *et al.*, 2021 ▸; McKinnon *et al.*, 2007 ▸; Spackman & Jayatilaka, 2009 ▸). This analysis, to some extent, also allows a quantitative comparison of the intermolecular contacts at varied temperature and pressure conditions. The different colours in the *d*_norm_ surfaces relate to the distances of all contacts and the sums of the van der Waals radii of relevant atoms (Fig. 4 ▸).

**Figure 5:**
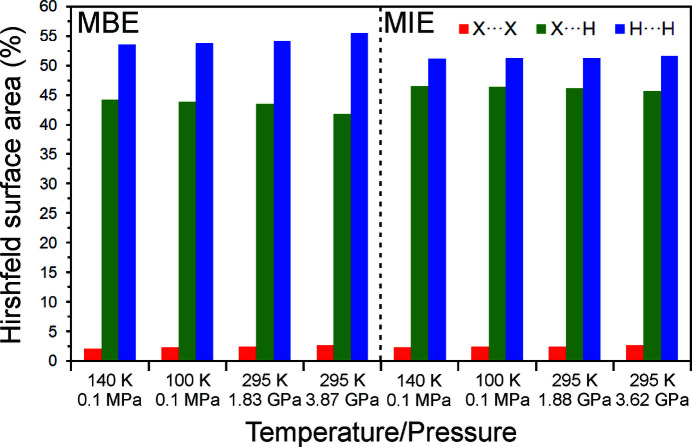
Distribution of *X*⋯*X*, *X*⋯H and H⋯H contacts, based on their relative contributions to the Hirshfeld surface area, for **MBE** and **MIE** at various selected temperature and pressure conditions (*X* = Br for **MBE**, and *X* = I for **MIE**).

As mentioned above, it can be seen that the shortest, relative to their van der Waals radii of interacting atoms, are the *X*⋯*X* and *X*⋯H interactions, while the longest inter­atomic distances have been found for the H⋯H contacts. The slightly different behaviour for **MBE** and **MIE** is shown in the Hirshfeld surface fingerprint plots. They present *d*_e_ as a function of *d*_i_ and also visualize the relative contributions of all types of contacts to the Hirshfeld surfaces (Figs. 5 ▸, S8 and S9). These two-dimensional diagrams show the general typical differences between the investigated structures: (i) the relatively shorter contacts for **MBE** than for **MIE** (the van der Waals radius for Br is smaller than for I) and (ii) more compressed, and more symmetrical, fingerprint plots for **MBE** and **MIE** determined at high pressures, as their contacts are compressed too. Inspection of the decomposed fingerprint plots facilitates a simple comparison between **MBE** and **MIE** molecules in their crystal structures determined at various temperature and pressure conditions. Also, the changes in contact distances and the shape of the *X*⋯H and H⋯H regions should be noticed. The shortest *X*⋯*X* contacts comprise only, on average, ∼2% of the total Hirshfeld surface area for both **MBE** and **MIE** and their contributions slightly increase with lowering temperature and increasing pressure. The clearly higher average contributions of ∼45%, that are the lowest at 100 K and ∼3.7 GPa, are noted for the *X*⋯H contacts (Saha *et al.*, 2018 ▸). The longest H⋯H contacts dominate the Hirshfeld surfaces, for both **MBE** and **MIE** at all thermodynamic conditions, with a percentage average contribution of ∼53%. Also, the contributions of those contacts slightly increase upon decreasing temperature and increasing pressure.

The observation of progressive shortening of all contact distances confirms their attractive nature, whereas the changes associated with contributions of particular groups of contacts to the Hirshfeld surface area suggest their hierarchical and competitive character.

## 4. Summary and conclusions

Bromo­ethane (**MBE**) and iodo­ethane (**MIE**) characterized by melting points of ∼160 K, with the lower melting point for **MBE** than for **MIE**, have been investigated at ambient-pressure low-temperature and ambient-temperature high-pressure conditions, starting with *in situ* crystallization of those liquids followed by single-crystal X-ray diffraction. The results of these studies, supported by the ambient-temperature compressibility and ambient-pressure thermoanalytical DSC measurements, demonstrate the clear similarities and only slight differences in the behaviour of these two isostructural crystals. Besides the close structural relationship both **MBE** and **MIE** show just one monoclinic phase that is stable down to 100 K/0.1 MPa and up to ∼ 295 K/3.7 GPa with no phase transition or symmetry change. These facts make them the perfect compounds for monitoring and analysing the influence of external low-temperature and high-pressure conditions on the simple molecular systems.

The study illustrates that the crystal structure of **MBE** is somewhat more close packed than its iodine analogue, **MIE**. This is related to the properties of different halogen atoms present in both crystal structures. Both decreasing temperature and increasing pressure, in the studied regimes, do not significantly affect the molecular dimensions, but clearly the intermolecular distances in both crystals are reduced. As a result the shorter and new intermolecular contacts are introduced, in particular, with compression of the crystals. The shortest type-I *X*⋯*X* (*X* = Br for **MBE**, and *X* = I for **MIE**) interactions, with average distances of ∼91% of the sums of the van der Waals radii of respective atoms have been found at the highest investigated pressures of ∼295 K/3.7 GPa. The distorted type-I *X*⋯H contacts, at the highest pressure of ∼295 K/3.7 GPa, are in a similar relation (93%) on average, of the sums of the van der Waals radii. The longest, mostly with the distances above the sum of the van der Waals radii, are the separation distances for H⋯H contacts.

The Hirshfeld surface analysis shows that the relative contributions of particular types of contacts are somewhat different for **MBE** and **MIE**, and slightly change upon decreasing temperature and increasing pressure confirming their attractive and hierarchical nature. The contribution of the strongest *X*⋯*X* contacts is as small as ∼2%, whereas the contribution of H⋯H contacts is as much as ∼53%. The input of those two types of contacts increase upon decreasing temperature and increasing pressure whereas the contributions of *X*⋯H contacts are gradually reduced to ∼45% at the highest pressure of ∼295 K/3.7 GPa. This indicates that both **MBE** and **MIE** molecules in their crystals, upon cooling or compression, are brought closer to each other and as a result the intermolecular distances get shorter without main changes in their mutual orientation. It is also worth mentioning that a detailed analysis of the crystal structures of relatively simple and analogous compounds together with monitoring their behaviour under temperature and pressure variations clearly contribute to the understanding of the noncovalent intermolecular interactions and their role on the fundamental processes of crystallization and solid-state structure formation. These along with the specific response of the crystals to the external forces could be used in the design of new materials through utilization of the crystal engineering approach.

## Supplementary Material

Crystal structure: contains datablock(s) global, MBE_140K, MBE_120K, MBE_100K, MBE_1.83GPa, MBE_2.13GPa, MBE_3.07GPa, MBE_3.87GPa, MIE_140K, MIE_120K, MIE_100K, MIE_1.88GPa, MIE_2.40GPa, MIE_3.18GPa, MIE_3.62GPa. DOI: 10.1107/S2052520622010149/aw5073sup1.cif

Structure factors: contains datablock(s) MBE_140K. DOI: 10.1107/S2052520622010149/aw5073MBE_140Ksup2.hkl

Structure factors: contains datablock(s) MBE_120K. DOI: 10.1107/S2052520622010149/aw5073MBE_120Ksup3.hkl

Structure factors: contains datablock(s) MBE_100K. DOI: 10.1107/S2052520622010149/aw5073MBE_100Ksup4.hkl

Structure factors: contains datablock(s) MBE_1.83GPa. DOI: 10.1107/S2052520622010149/aw5073MBE_1.83GPasup5.hkl

Structure factors: contains datablock(s) MBE_2.13GPa. DOI: 10.1107/S2052520622010149/aw5073MBE_2.13GPasup6.hkl

Structure factors: contains datablock(s) MBE_3.07GPa. DOI: 10.1107/S2052520622010149/aw5073MBE_3.07GPasup7.hkl

Structure factors: contains datablock(s) MBE_3.87GPa. DOI: 10.1107/S2052520622010149/aw5073MBE_3.87GPasup8.hkl

Structure factors: contains datablock(s) MIE_140K. DOI: 10.1107/S2052520622010149/aw5073MIE_140Ksup9.hkl

Structure factors: contains datablock(s) MIE_120K. DOI: 10.1107/S2052520622010149/aw5073MIE_120Ksup10.hkl

Structure factors: contains datablock(s) MIE_100K. DOI: 10.1107/S2052520622010149/aw5073MIE_100Ksup11.hkl

Structure factors: contains datablock(s) MIE_1.88GPa. DOI: 10.1107/S2052520622010149/aw5073MIE_1.88GPasup12.hkl

Structure factors: contains datablock(s) MIE_2.40GPa. DOI: 10.1107/S2052520622010149/aw5073MIE_2.40GPasup13.hkl

Structure factors: contains datablock(s) MIE_3.18GPa. DOI: 10.1107/S2052520622010149/aw5073MIE_3.18GPasup14.hkl

Structure factors: contains datablock(s) MIE_3.62GPa. DOI: 10.1107/S2052520622010149/aw5073MIE_3.62GPasup15.hkl

Tables S1-S14 and Figs S1-S9. DOI: 10.1107/S2052520622010149/aw5073sup16.pdf

Supporting information file. DOI: 10.1107/S2052520622010149/aw5073MBE_1.83GPasup17.cml

Supporting information file. DOI: 10.1107/S2052520622010149/aw5073MIE_1.88GPasup18.cml

CCDC references: 2213873, 2213874, 2213875, 2213876, 2213877, 2213878, 2213879, 2213880, 2213881, 2213882, 2213883, 2213884, 2213885, 2213886

## Figures and Tables

**Table 1:** Selected crystal data for **MBE** at various temperature and pressure conditions For all experiments: C_2_H_5_Br, *M*_r_ = 108.96, monoclinic crystal system, space group *P*2_1_/*n* and *Z* = 4.

Temperature	140.0 (1) K	120.0 (1) K	100.0 (1) K	295 (2) K	295 (2) K	295 (2) K	295 (2) K
Pressure	0.1 MPa	0.1 MPa	0.1 MPa	1.83 (2) GPa	2.13 (2) GPa	3.07 (2) GPa	3.87 (2) GPa
*a* (Å)	5.5329 (5)	5.5204 (5)	5.5087 (4)	5.2831 (6)	5.2524 (6)	5.1730 (8)	5.107 (3)
*b* (Å)	9.9018 (10)	9.8941 (8)	9.8814 (8)	9.4910 (9)	9.4479 (12)	9.3305 (18)	9.206 (7)
*c* (Å)	7.0147 (7)	6.9655 (6)	6.9315 (6)	6.5957 (6)	6.5350 (6)	6.3822 (9)	6.285 (3)
β (°)	100.309 (9)	100.340 (9)	100.379 (8)	100.623 (11)	100.664 (10)	100.777 (17)	101.08 (6)
*V* (Å^3^)	378.10 (6)	374.27 (6)	371.13 (5)	325.05 (6)	318.69 (6)	302.61 (9)	290.0 (3)
*R*[*F* ^2^ > 2σ(*F* ^2^)]	0.0211	0.0220	0.0211	0.0163	0.0189	0.0314	0.0584
*wR*(*F* ^2^)	0.0433	0.0506	0.0484	0.0425	0.0521	0.0892	0.1346

**Table 2:** Selected crystal data for **MIE** at various temperature and pressure conditions For all experiments: C_2_H_5_I, *M*_r_ = 155.96, monoclinic crystal system, space group *P*2_1_/*n* and *Z* = 4.

Temperature	140.0 (1) K	120.0 (1) K	100.0 (1) K	295 (2) K	295 (2) K	295 (2) K	295 (2) K
Pressure	0.1 MPa	0.1 MPa	0.1 MPa	1.88 (2) GPa	2.40 (2) GPa	3.18 (2) GPa	3.62 (2) GPa
*a* (Å)	5.85463 (16)	5.83962 (16)	5.82719 (15)	5.5767 (13)	5.5292 (9)	5.4696 (8)	5.4335 (11)
*b* (Å)	10.1532 (3)	10.1442 (3)	10.1348 (2)	9.7073 (6)	9.6336 (7)	9.5403 (6)	9.4863 (7)
*c* (Å)	7.2722 (2)	7.2368 (2)	7.2028 (2)	6.8424 (3)	6.7597 (2)	6.6492 (6)	6.5840 (4)
β (°)	102.701 (3)	102.705 (3)	102.711 (3)	102.262 (10)	102.209 (8)	102.280 (13)	102.191 (12)
*V* (Å^3^)	421.71 (2)	418.20 (2)	414.96 (2)	361.96 (9)	351.92 (6)	339.03 (6)	331.71 (8)
*R*[*F* ^2^ > 2σ(*F* ^2^)]	0.0119	0.0115	0.0110	0.0256	0.0375	0.0352	0.0263
*wR*(*F* ^2^)	0.0272	0.0254	0.0231	0.0685	0.1378	0.0969	0.0700

**Table 3:** Dimensions (Å, °) of the shortest intermolecular contacts Br⋯Br and Br⋯H for **MBE** at various temperature and pressure conditions, compared to those commensurate with the sums of the van der Waals radii of respective atoms at 295 (2) K/3.87 (2) GPa

Temperature	140.0 (1) K	120.0 (1) K	100.0 (1) K	295 (2) K	295 (2) K	295 (2) K	295 (2) K
Pressure	0.1 MPa	0.1 MPa	0.1 MPa	1.83 (2) GPa	2.13 (2) GPa	3.07 (2) GPa	3.87 (2) GPa
Br1⋯Br1^i^	3.8860 (7)	3.8458 (6)	3.8155 (6)	3.6040 (9)	3.5606 (8)	3.465 (2)	3.393 (5)
C1—Br1⋯Br1^i^	156.63 (8)	157.40 (8)	157.79 (8)	157.91 (11)	158.13 (10)	159.3 (3)	161.4 (5)
Br1⋯Br1^i^—C1^i^	156.63 (8)	157.40 (8)	157.79 (8)	157.91 (11)	158.13 (10)	159.3 (3)	161.4 (5)
Br1⋯H11^ii^	3.24	3.24	3.23	3.05	3.02	2.96	2.96
C1—Br1⋯H11^ii^	139	138	138	135	134	133	132
Br1⋯H11^ii^–C1^ii^	136	136	136	136	136	136	132
Br1⋯H12^iii^	3.37	3.33	3.31	3.11	3.08	3.01	2.97
C1—Br1⋯H12^iii^	91	91	91	90	90	89	91
Br1⋯H12^iii^—C1^iii^	138	138	138	137	137	135	134
Br1⋯H22^iv^	3.30	3.27	3.25	3.06	3.04	3.02	3.05
C1—Br1⋯H22^iv^	109	109	109	113	112	111	106
Br1⋯H22^iv^—C2^iv^	138	139	138	141	140	135	124
Br1⋯H23^v^	3.14	3.14	3.14	2.99	2.95	2.84	2.83
C1—Br1⋯H23^v^	135	134	133	132	133	134	137
Br1⋯H23^v^—C2^v^	163	160	160	151	155	162	165

Symmetry codes: (i) 1 − *x*, 2 − *y*, 2 − *z*; (ii) 1 + *x*, *y*, *z*; (iii) ½ + *x*, 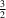 − *y*, ½ + *z*; (iv) −*x*, 2 − *y*, 2 − *z*; (v) ½ − *x*, ½ + *y*, 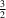 − *z*.

**Table 4:** Dimensions (Å, °) of the shortest intermolecular contacts I⋯I and I⋯H for **MIE** at various temperature and pressure conditions, compared to those commensurate with the sums of the van der Waals radii of respective atoms at 295 (2) K/3.62 (2) GPa

Temperature	140.0 (1) K	120.0 (1) K	100.0 (1) K	295 (2) K	295 (2) K	295 (2) K	295 (2) K
Pressure	0.1 MPa	0.1 MPa	0.1 MPa	1.88 (2) GPa	2.40 (2) GPa	3.18 (2) GPa	3.62 (2) GPa
I1⋯I1^i^	4.0008 (3)	3.9770 (3)	3.9564 (3)	3.7574 (13)	3.7072 (18)	3.639 (2)	3.6061 (15)
C1—I1⋯I1^i^	154.22 (6)	154.63 (5)	154.93 (5)	154.62 (15)	154.9 (3)	155.5 (3)	155.5 (2)
I1⋯I1^i^—C1^i^	154.22 (6)	154.63 (5)	154.93 (5)	154.62 (15)	154.9 (3)	155.5 (3)	155.5 (2)
I1⋯H11^ii^	3.39	3.38	3.37	3.17	3.16	3.08	3.06
C1—I1⋯H11^ii^	138	138	137	134	134	133	132
I1⋯H11^ii^—C1^ii^	138	138	138	138	138	138	138
I1⋯H12^iii^	3.39	3.37	3.35	3.13	3.09	3.03	2.99
C1—I1⋯H12^iii^	87	87	87	86	86	86	86
I1⋯H12^iii^—C1^iii^	142	142	142	142	141	141	141
I1⋯H22^iv^	3.42	3.40	3.39	3.19	3.15	3.13	3.08
C1—I1⋯H22^iv^	108	108	108	111	112	110	112
I1⋯H22^iv^—C2^iv^	140	140	140	141	142	137	140
I1⋯H23^v^	3.31	3.30	3.29	3.09	3.06	2.99	2.98
C1—I1⋯H23^v^	139	139	138	139	138	140	138
I1⋯H23^v^—C2^v^	164	164	164	160	157	165	159

Symmetry codes: (i) 1 − *x*, 2 − *y*, 2 − *z*; (ii) 1 + *x*, *y*, *z*; (iii) ½ + *x*, 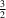 − *y*, ½ + *z*; (iv) −*x*, 2 − *y*, 2 − *z*; (v) ½ − *x*, ½ + *y*, 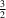 − *z*.
