# Supplementary material for: Evolution of intermolecular contacts with temperature and pressure in bromoethane and iodo­ethane – a comparative study
Source: Acta Crystallogr B Struct Sci Cryst Eng Mater. 2022 Nov 9;78(Pt 6):868–75. doi: 10.1107/S2052520622010149 (PMC9728020; doi:10.1107/S2052520622010149)
Supplement: Supplementary file 16 [file b-78-00868-sup16.pdf]

STRUCTURAL SCIENCE
CRYSTAL ENGINEERING
MATERIALS

Volume 78 (2022)

Supporting information for article:

Evolution of intermolecular contacts with temperature and pressure in bromoethane and iodoethane – a comparative study

Maciej Bujak, Anna Olejniczak and Marcin Podsiadło

Table of Contents

Table S1. Selected crystal data and structure determination summary for **MBE** at 140, 120 and 100 K……….…………… 2

Table S2. Selected crystal data and structure determination summary for **MIE** at 140, 120 and 100 K………………..…….. 2

Table S3. Selected crystal data and structure determination summary for **MBE** at 1.83, 2.13, 3.07 and 3.87 GPa…............... 3

Table S4. Selected crystal data and structure determination summary for **MIE** at 1.88, 2.40, 3.18 and 3.62 GPa…………….. 3

Table S5. Coefficients of thermal expansion for **MBE**, related to crystallographic axes, calculated in the range between 100 and 140 K…………………………………………………………………………………………………... 4

Table S6. Coefficients of thermal expansion for **MIE**, related to crystallographic axes, calculated in the range between 100 and 140 K…………………………………………………………………………………………………... 4

Table S7. Coefficients of compressibility for **MBE**, related to crystallographic axes, calculated in the range between 1.83 and 3.87 GPa along with Birch-Murnaghan coefficients……………………………………………………………….. 4

Table S8. Coefficients of compressibility for **MIE**, related to crystallographic axes, calculated in the range between 1.88 and 3.62 GPa along with Birch-Murnaghan coefficients……………………………………………………………….. 4

Table S9. Selected bond lengths (Å) and bond angles (°) for **MBE** at variable temperature and pressure conditions..……… 5

Table S10. Selected bond lengths (Å) and bond angles (°) for **MIE** at variable temperature and pressure conditions.....…… 5

Table S11. Dimensions (Å, °) of the shortest intermolecular contacts for **MBE** at variable temperature and pressure conditions, compared to those commensurate with the sums of the van der Waals radii of respective atoms at 295 K/3.87 GPa. 6

Table S12. Dimensions (Å, °) of the shortest intermolecular contacts for **MIE** at variable temperature and pressure conditions, compared to those commensurate with the sums of the van der Waals radii of respective atoms at 295 K/3.62 GPa……………. 8

Table S13. Hydrogen bonds geometries (Å, °) for **MBE** at variable temperature and pressure conditions, compared to those commensurate with the sums of the van der Waals radii of Br and H atoms at 295 K/3.87 GPa…….…………………….. 9

Table S14. Hydrogen bonds geometries (Å, °) for **MIE** at variable temperature and pressure conditions, compared to those commensurate with the sums of the van der Waals radii of I and H atoms at 295 K/3.62 GPa…….……………………….. 10

Figure S1. Stages of isochoric, with decreasing temperature, growth of the **MBE** single crystal in a diamond-anvil cell initially pressurized, at ambient temperature to *ca.* 2 GPa, started at 350(3) K (a), leading to the sample fully filling the high-pressure chamber at 1.83 GPa (f). The pressure chamber is 0.37 mm in diameter. The ruby chip, for pressure calibration, is located at the down-left side of the high-pressure chamber….………………………………………….….. 11

Figure S2. Stages of isochoric, with decreasing temperature, growth of the **MIE** single crystal in a diamond-anvil cell initially pressurized, at ambient temperature to *ca.* 2 GPa, started at 365(3) K (a), leading to the sample fully filling the high-pressure chamber at 1.88 GPa (f). The pressure chamber is 0.37 mm in diameter. The ruby chip, for pressure calibration, is located at the middle-left side of the high-pressure chamber….……………………………………….… 11

Figure S3. Comparison of the average ADP values for C1, C2 and X (X = Br for **MBE** and X = I for **MIE**) atoms as well as unit-volume changes at variable investigated temperature and pressure points. The error bars for ADP, at different pressure points, are indicated. The lines joining the points are guides for the eye only……………..……………………….. 12

Figure S4. DSC cooling and heating runs measured at the rate of 10 K·min^{-1}, at ambient pressure, for **MBE**….……..……. 13

Figure S5. DSC cooling and heating runs measured at the rate of 10 K·min^{-1}, at ambient pressure, for **MIE**………...…….. 13

Figure S6. Molecular volume of **MBE** at room temperature as a function of pressure measured in the piston-and-cylinder press (green diamonds). The volumes measured at high pressure (red triangles) and low temperature (blue circles) by single-crystal X-ray diffraction are indicated. The red dashed line is for guiding the eye only………………………………. 14

Figure S7. Molecular volume of **MIE** at room temperature as a function of pressure measured in the piston-and-cylinder press (green diamonds). The volumes measured at high pressure (red triangles) and low temperature (blue circles) by single-crystal X-ray diffraction are indicated. The red dashed line is for guiding the eye only………………………………. 14

Figure S8. Two-dimensional fingerprint plots of **MBE** determined at: 140 K/0.1 MPa (a); 100 K/0.1 MPa (b); 295 K/1.83 GPa (c) and 295 K/3.87 GPa (d). The Br···Br, Br···H and H···H contacts are highlighted……………………...………… 15

Figure S9. Two-dimensional fingerprint plots of **MIE** determined at: 140 K/0.1 MPa (a); 100 K/0.1 MPa (b); 295 K/1.88 GPa (c) and 295 K/3.62 GPa (d). The I···I, I···H and H···H contacts are highlighted………………………………………… 16

References (SI)…………………………………………………………………………………………..……… 17

Table S1. Selected crystal data and structure determination summary for **MBE** at 140, 120 and 100 K.

temperature (K)	140.0(1)	120.0(1)	100.0(1)
pressure (MPa)	0.1	0.1	0.1
formula	C_2H_5Br	C_2H_5Br	C_2H_5Br
M_r	108.96	108.96	108.96
crystal size (mm)	0.1 × 0.1 × 0.1	0.1 × 0.1 × 0.1	0.1 × 0.1 × 0.1
crystal system	monoclinic	monoclinic	monoclinic
space group, *Z*, *Z'*	$P2_1/n$, 4, 1	$P2_1/n$, 4, 1	$P2_1/n$, 4, 1
a (Å)	5.5329(5)	5.5204(5)	5.5087(4)
b (Å)	9.9018(10)	9.8941(8)	9.8814(8)
c (Å)	7.0147(7)	6.9655(6)	6.9315(6)
β (deg)	100.309(9)	100.340(9)	100.379(8)
V (Å^3)	378.10(6)	374.27(6)	371.13(5)
ρ (g/cm^3)	1.914	1.934	1.950
μ (mm^{-1})	10.607	10.715	10.806
θ range (deg)	3.60 - 27.49	3.62 - 27.49	3.63 - 27.49
index ranges	$-7 \le h \le 7$	$-7 \le h \le 7$	$-7 \le h \le 7$
	$-12 \le k \le 12$	$-12 \le k \le 12$	$-12 \le k \le 12$
	$-9 \le l \le 9$	$-9 \le l \le 9$	$-8 \le l \le 8$
reflns collected	5889	5791	5716
R_{int}	0.0529	0.0491	0.0485
data [$I > 2\sigma(I)$]	756	759	770
data/parameters	872/30	861/29	850/29
GOF on F^2	1.044	1.094	1.082
R_1 [$F^2 > 2\sigma(F^2)$]	0.0211	0.0220	0.0211
R_1 (all data)	0.0272	0.0277	0.0252
wR_2 (all data)	0.0433	0.0506	0.0484
lrgst diff peak (e/Å^3)	0.382	0.650	0.703
lrgst diff hole (e/Å^3)	–0.337	–0.557	–0.544

Table S2. Selected crystal data and structure determination summary for **MIE** at 140, 120 and 100 K.

temperature (K)	140.0(1)	120.0(1)	100.0(1)
pressure (MPa)	0.1	0.l	0.1
formula	C_2H_5I	C_2H_5I	C_2H_5I
M_r	155.96	155.96	155.96
crystal size (mm)	0.2 × 0.2 × 0.2	0.2 × 0.2 × 0.2	0.2 × 0.2 × 0.2
crystal system	monoclinic	monoclinic	monoclinic
space group, *Z*, *Z'*	$P2_1/n$, 4, 1	$P2_1/n$, 4, 1	$P2_1/n$, 4, 1
a (Å)	5.85463(16)	5.83962(16)	5.82719(15)
b (Å)	10.1532(3)	10.1442(3)	10.1348(2)
c (Å)	7.2722(2)	7.2368(2)	7.20284(19)
β (deg)	102.701(3)	102.705(3)	102.711(3)
V (Å^3)	421.71(2)	418.198(19)	414.956(18)
ρ (g/cm^3)	2.456	2.477	2.496
μ (mm^{-1})	7.357	7.419	7.477
θ range (deg)	3.50 - 27.49	3.52 - 27.48	3.53 - 27.50
index ranges	$-7 \le h \le 7$	$-7 \le h \le 7$	$-7 \le h \le 7$
	$-13 \le k \le 13$	$-13 \le k \le 13$	$-13 \le k \le 13$
	$-9 \le l \le 9$	$-9 \le l \le 9$	$-9 \le l \le 9$
reflns collected	7391	7329	7277
R_{int}	0.0173	0.0178	0.0164
data [$I > 2\sigma(I)$]	952	949	951
data/parameters	969/30	962/30	959/30
GOF on F^2	1.246	1.272	1.314
R_1 [$F^2 > 2\sigma(F^2)$]	0.0119	0.0115	0.0110
R_1 (all data)	0.0123	0.0119	0.0112
wR_2 (all data)	0.0272	0.0254	0.0231
lrgst diff peak (e/Å^3)	0.348	0.304	0.353
lrgst diff hole (e/Å^3)	–0.282	–0.269	–0.241

Table S3. Selected crystal data and structure determination summary for **MBE** at 1.83, 2.13, 3.07 and 3.87 GPa.

temperature (K)	295(2)	295(2)	295(2)	295(2)
pressure (GPa)	1.83(2)	2.13(2)	3.07(2)	3.87(2)
formula	C_2H_5Br	C_2H_5Br	C_2H_5Br	C_2H_5Br
M_r	108.96	108.96	108.96	108.96
crystal size (mm)	0.37 × 0.37 × 0.06	0.37 × 0.37 × 0.06	0.37 × 0.37 × 0.05	0.37 × 0.37 × 0.04
crystal system	monoclinic	monoclinic	monoclinic	monoclinic
space group, *Z*, *Z'*	$P2_1/n$, 4, 1	$P2_1/n$, 4, 1	$P2_1/n$, 4, 1	$P2_1/n$, 4, 1
a (Å)	5.2831(6)	5.2524(6)	5.1730(8)	5.107(3)
b (Å)	9.4910(9)	9.4479(12)	9.3305(18)	9.206(7)
c (Å)	6.5957(6)	6.5350(6)	6.3822(9)	6.285(3)
β (deg)	100.623(11)	100.664(10)	100.777(17)	101.08(6)
V (Å^3)	325.05(6)	318.69(6)	302.61(9)	290.0(3)
ρ (g/cm^3)	2.227	2.271	2.392	2.496
μ (mm^{-1})	12.338	12.584	13.253	13.831
θ range (deg)	4.47 - 26.77	4.50 - 27.15	4.57 - 26.83	4.63 - 26.54
index ranges	$-5 \le h \le 5$	$-6 \le h \le 5$	$-5 \le h \le 5$	$-5 \le h \le 5$
	$-9 \le k \le 9$	$-9 \le k \le 9$	$-9 \le k \le 9$	$-8 \le k \le 8$
	$-7 \le l \le 7$	$-7 \le l \le 7$	$-7 \le l \le 7$	$-7 \le l \le 7$
reflns collected	1621	1588	1501	1363
R_{int}	0.0184	0.0174	0.0235	0.0438
data [$I > 2\sigma(I)$]	258	266	244	200
data/parameters	277/29	277/29	269/29	252/29
GOF on F^2	1.245	1.150	1.174	1.226
R_1 [$F^2 > 2\sigma(F^2)$]	0.0163	0.0189	0.0314	0.0584
R_1 (all data)	0.0183	0.0194	0.0358	0.0747
wR_2 (all data)	0.0425	0.0521	0.0892	0.1346
lrgst diff peak (e/Å^3)	0.149	0.286	0.397	0.846
lrgst diff hole (e/Å^3)	–0.151	–0.266	–0.435	–0.534

Table S4. Selected crystal data and structure determination summary for **MIE** at 1.88, 2.40, 3.18 and 3.62 GPa.

temperature (K)	295(2)	295(2)	295(2)	295(2)
pressure (GPa)	1.88(2)	2.40(2)	3.18(2)	3.62(2)
formula	C_2H_5I	C_2H_5I	C_2H_5I	C_2H_5I
M_r	155.96	155.96	155.96	155.96
crystal size (mm)	0.37 × 0.37 × 0.21	0.37 × 0.37 × 0.19	0.37 × 0.37 × 0.16	0.37 × 0.37 × 0.14
crystal system	monoclinic	monoclinic	monoclinic	monoclinic
space group, *Z*, *Z'*	$P2_1/n$, 4, 1	$P2_1/n$, 4, 1	$P2_1/n$, 4, 1	$P2_1/n$, 4, 1
a (Å)	5.5767(13)	5.5292(9)	5.4696(8)	5.4335(11)
b (Å)	9.7073(6)	9.6336(7)	9.5403(6)	9.4863(7)
c (Å)	6.8424(3)	6.7597(2)	6.6492(6)	6.5840(4)
β (deg)	102.262(10)	102.209(8)	102.280(13)	102.191(12)
V (Å^3)	361.96(9)	351.92(6)	339.03(6)	331.71(8)
ρ (g/cm^3)	2.862	2.944	3.056	3.123
μ (mm^{-1})	8.571	8.816	9.151	9.353
θ range (deg)	4.78 - 26.77	4.32 - 26.91	4.37 - 26.88	4.93 - 26.56
index ranges	$-4 \le h \le 4$	$-4 \le h \le 4$	$-5 \le h \le 5$	$-4 \le h \le 4$
	$-11 \le k \le 11$	$-10 \le k \le 10$	$-10 \le k \le 10$	$-10 \le k \le 10$
	$-8 \le l \le 8$	$-8 \le l \le 8$	$-7 \le l \le 7$	$-8 \le l \le 8$
reflns collected	1671	1557	1416	1394
R_{int}	0.0254	0.0345	0.0424	0.0216
data [$I > 2\sigma(I)$]	293	294	271	263
data/parameters	299/30	301/29	275/30	270/30
GOF on F^2	1.256	1.380	1.324	1.357
R_1 [$F^2 > 2\sigma(F^2)$]	0.0256	0.0375	0.0352	0.0263
R_1 (all data)	0.0268	0.0379	0.0355	0.0270
wR_2 (all data)	0.0685	0.1378	0.0969	0.0700
lrgst diff peak (e/Å^3)	0.474	0.743	0.596	0.407
lrgst diff hole (e/Å^3)	–0.412	–0.680	–0.541	–0.368

Table S5. Coefficients of thermal expansion for **MBE**, related to crystallographic axes**,** calculated in the range between 100 and 140 K (Cliffe & Goodwin, 2012).

axes	α(MK^{-1})	σα(MK^{-1})	direction		
			a	*b*	*c*
X_1	51.6121	2.9816	0.0000	1.0000	–0.0000
X_2	109.7853	0.8790	–0.9997	0.0000	0.0246
X_3	305.7203	12.7487	0.2554	-0.0000	0.9668
V	470.8930	10.9727			

Table S6. Coefficients of thermal expansion for **MIE,** related to crystallographic axes, calculated in the range between 100 and 140 K (Cliffe & Goodwin, 2012).

axes	α(MK^{-1})	σα(MK^{-1})	direction		
			a	*b*	*c*
X_1	45.3882	0.2326	–0.0000	1.0000	–0.0000
X_2	116.6327	2.6074	–0.9973	0.0000	–0.0734
X_3	242.8181	1.1337	0.1607	–0.0000	0.9870
V	406.8429	3.7391			

Table S7. Coefficients of compressibility for **MBE**, related to crystallographic axes, calculated in the range between 1.83 and 3.87 GPa along with Birch-Murnaghan coefficients (Cliffe & Goodwin, 2012).

axes	K(TPa^{-1})	σK(TPa^{-1})	direction			empirical parameters			
			a	*b*	*c*	ε_0	λ	*Pc*	ν
X_1	18.6194	0.0000	0.2436	–0.0000	0.9699	7.7143e-02	–7.5221e-02	0.7733	0.4572
X_2	14.6894	0.0000	–0.9999	0.0000	0.0139	–3.0202e-05	–1.7154e-02	1.8300	0.9119
X_3	16.5787	0.0000	0.0000	1.0000	–0.0000	4.0142e-02	–5.6481e-04	–5.3042	2.1743
V	52.5895	1.0740							

Birch-Murnaghan coefficients

	B_0 (GPa)	σB_0 (GPa)	V_0 (Å^3)	σV_0 (Å^3)	*B*’	σ*B*’	P_c (GPa)
2nd	7.8627	0.3415	384.2440	2.6464	4	n/a	0
3rd	12.2015	2.0667	369.4359	5.4695	2.5288	0.5983	0

Table S8. Coefficients of compressibility for **MIE**, related to crystallographic axes, calculated in the range between 1.88 and 3.62 GPa along with Birch-Murnaghan coefficients (Cliffe & Goodwin, 2012).

axes	K(TPa^{-1})	σK(TPa^{-1})	direction			empirical parameters			
			a	*b*	*c*	ε_0	λ	*Pc*	ν
X_1	20.6062	0.0000	0.1493	–0.0000	0.9888	–6.8206e-05	–2.2172e-02	1.8800	0.9536
X_2	13.5386	0.0000	–0.9972	0.0000	–0.0745	–5.4125e-05	–1.5029e-02	1.8800	0.9342
X_3	11.9635	0.0000	0.0000	1.0000	0.0000	–5.2635e-05	–1.3573e-02	1.8800	0.9209
V	47.7304	0.7445							

Birch-Murnaghan coefficients

	B_0 (GPa)	σB_0 (GPa)	V_0 (Å^3)	σV_0 (Å^3)	*B*’	σ*B*’	P_c (GPa)
2nd	10.0639	0.3654	416.9624	2.1201	4	n/a	0
3rd	14.7437	2.9115	404.9298	6.0302	2.3771	0.8760	0

Table S9. Selected bond lengths (Å) and bond angles (°) for **MBE** at variable temperature and pressure conditions.

temperature	140 K	120 K	100 K	295 K	295 K	295 K	295 K
pressure	0.1 MPa	0.1 MPa	0.1 MPa	1.83 GPa	2.13 GPa	3.07 GPa	3.87 GPa
Br1–C1	1.955(3)	1.959(3)	1.964(3)	1.955(4)	1.958(4)	1.954(9)	1.914(18)
C1–C2	1.485(3)	1.490(4)	1.492(3)	1.491(5)	1.476(5)	1.506(13)	1.56(3)
Br1–C1–C2	111.72(17)	111.59(18)	111.40(17)	111.5(3)	111.6(2)	111.2(6)	109.5(13)

Table S10. Selected bond lengths (Å) and bond angles (°) for **MIE** at variable temperature and pressure conditions.

temperature	140 K	120 K	100 K	295 K	295 K	295 K	295 K
pressure	0.1 MPa	0.1 MPa	0.1 MPa	1.88 GPa	2.40 GPa	3.18 GPa	3.62 GPa
I1–C1	2.1572(19)	2.1603(18)	2.1612(18)	2.155(12)	2.129(15)	2.151(15)	2.144(13)
C1–C2	1.495(3)	1.500(3)	1.502(3)	1.482(9)	1.494(14)	1.508(15)	1.499(10)
I1–C1–C2	112.33(12)	112.10(12)	112.04(11)	112.5(6)	113.5(9)	112.0(10)	112.1(7)

Table S11. Dimensions (Å, °) of the shortest intermolecular contacts for **MBE** at variable temperature and pressure conditions, compared to those commensurate with the sums of the van der Waals radii of respective atoms at 295 K/3.87 GPa (Bondi, 1964).

temperature<br>pressure	140 K<br>0.1 MPa	120 K<br>0.1 MPa	100 K<br>0.1 MPa	295 K<br>1.83 GPa	295 K<br>2.13 GPa	295 K<br>3.07 GPa	295 K<br>3.87 GPa
Br1···Br1^{i}	3.8860(7)	3.8458(6)	3.8155(6)	3.6040(9)	3.5606(8)	3.465(2)	3.393(5)
C1–Br1···Br1^{i}	156.63(8)	157.40(8)	157.79(8)	157.91(11)	158.13(10)	159.3(3)	161.4(5)
Br1···Br1^{i}–C1^{i}	156.63(8)	157.40(8)	157.79(8)	157.91(11)	158.13(10)	159.3(3)	161.4(5)
C1–Br1···Br1^{i}–C1^{i}	180.00	180.00	180.00	180.00	180.00	180.0	180.0
Br1···H11^{ii}	3.24	3.24	3.23	3.05	3.02	2.96	2.96
C1–Br1···H11^{ii}	139	138	138	135	134	133	132
Br1···H11^{ii}–C1^{ii}	136	136	136	136	136	136	132
C1–Br1···H11^{ii}–C1^{ii}	–62	–64	–65	–70	–70	–72	–79
H11···Br1^{iii}	3.24	3.24	3.23	3.05	3.02	2.96	2.96
C1–H11···Br1^{iii}	136	136	136	136	136	136	132
H11···Br1^{iii}–C1^{iii}	139	138	138	135	134	133	132
C1–H11···Br1^{iii}–C1^{iii}	–62	–64	–65	–70	–70	–72	–79
Br1···H12^{iv}	3.45	3.45	3.45	3.24	3.22	3.13	3.04
C1–Br1···H12^{iv}	87	87	87	88	88	88	85
Br1···H12^{iv}–C1^{iv}	122	121	121	118	117	117	115
C1–Br1···H12^{iv}–C1^{iv}	–69	–69	–69	–69	–69	–69	–70
H12···Br1^{iv}	3.45	3.45	3.45	3.24	3.22	3.13	3.04
C1–H12···Br1^{iv}	122	121	121	118	117	117	115
H12···Br1^{iv}–C1^{iv}	87	87	87	88	88	88	85
C1–H12···Br1^{iv}–C1^{iv}	69	69	69	69	69	69	70
Br1···H12^{v}	3.37	3.33	3.31	3.11	3.08	3.01	2.97
C1–Br1···H12^{v}	91	91	91	90	90	89	91
Br1···H12^{v}–C1^{v}	138	138	138	137	137	135	134
C1–Br1···H12^{v}–C1^{v}	–97	–96	–96	–95	–95	–96	–95
H12···Br1^{vi}	3.37	3.33	3.31	3.11	3.08	3.01	2.97
C1–H12···Br1^{vi}	138	138	138	137	137	135	134
H12···Br1^{vi}–C1^{vi}	91	91	91	90	90	89	91
C1–H12···Br1^{vi}–C1^{vi}	97	96	96	95	95	96	95
Br1···H21^{ii}	3.47	3.48	3.48	3.42	3.35	3.22	2.91
C1–Br1···H21^{ii}	111	111	110	106	106	105	108
Br1···H21^{ii}–C2^{ii}	112	110	110	101	104	109	126
C1–Br1···H21^{ii}–C2^{ii}	–178	–177	–178	–176	–177	180	167
H21···Br1^{iii}	3.47	3.48	3.48	3.42	3.35	3.22	2.91
C2–H21···Br1^{iii}	112	110	110	101	104	109	126
H21···Br1^{iii}–C1^{iii}	111	111	110	106	106	105	108
C2–H21···Br1^{iii}–C1^{iii}	–178	–177	–178	–176	–177	180	167
Br1···H22^{ix}	3.30	3.27	3.25	3.06	3.04	3.02	3.05
C1–Br1···H22^{ix}	109	109	109	113	112	111	106
Br1···H22^{ix}–C2^{ix}	138	139	138	141	140	135	124
C1–Br1···H22^{ix}–C2^{ix}	96	95	95	82	88	100	123
H22···Br1^{ix}	3.30	3.27	3.25	3.06	3.04	3.02	3.05
C2–H22···Br1^{ix}	138	139	138	141	140	135	124
H22···Br1^{ix}–C1^{ix}	109	109	109	113	112	111	106
C2–H22···Br1^{ix}–C1^{ix}	–96	–95	–95	–82	–88	–100	–123
Br1···H22^{x}	3.42	3.40	3.38	3.20	3.17	3.07	3.04
C1–Br1···H22^{x}	78	77	77	73	73	75	79
Br1···H22^{x}–C2^{x}	122	121	121	118	118	119	116
C1–Br1···H22^{x}–C2^{x}	92	94	94	104	100	91	68
H22···Br1^{xi}	3.42	3.40	3.38	3.20	3.17	3.07	3.04
C2–H22···Br1^{xi}	122	121	121	118	118	119	116
H22···Br1^{xi}–C1^{xi}	78	77	77	73	73	75	79
C2–H22···Br1^{xi}–C1^{xi}	–92	–94	–94	–104	–100	–91	–68
Br1···H23^{xii}	3.14	3.14	3.14	2.99	2.95	2.84	2.83
C1–Br1···H23^{xii}	135	134	133	132	133	134	137
Br1···H23^{xii}–C2^{xii}	163	160	160	151	155	162	165
C1–Br1···H23^{xii}–C2^{xii}	166	166	166	169	167	156	72

H23···Br1^{xiii}	3.14	3.14	3.14	2.99	2.95	2.84	2.83
C2–H23···Br1^{xiii}	163	160	160	151	155	162	165
H23···Br1^{xiii}–C1^{xiii}	135	134	133	132	133	134	137
C2–H23···Br1^{xiii}–C1^{xiii}	166	166	166	169	167	156	72

Symmetry codes: (i) 1 – *x* , 2 – *y*, 2 – *z*; (ii) 1 + *x*, *y*, *z*; (iii) –1 + *x*, *y*, *z*; (iv) –*x*, 2 – *y*, 1 – *z*; (v) 1/2 + *x*, 3/2 – *y*, 1/2 + *z*; (vi) –1/2 + *x*, 3/2 – *y*, –1/2 + *z*; (ix) –*x*, 2 – *y*, 2 – *z*; (x) 1/2 + *x*, 3/2 – *y*, –1/2 + *z*; (xi) –1/2 + *x*, 3/2 – *y*, 1/2 + *z*; (xii) 1/2 – *x*, 1/2 + *y*, 3/2 – *z*; (xiii) 1/2 – *x*, –1/2 + *y*, 3/2 – *z*.

Table S12. Dimensions (Å, °) of the shortest intermolecular contacts for **MIE** at variable temperature and pressure conditions, compared to those commensurate with the sums of the van der Waals radii of respective atoms at 295 K/3.62 GPa (Bondi, 1964).

temperature<br>pressure	140 K<br>0.1 MPa	120 K<br>0.1 MPa	100 K<br>0.1 MPa	295 K<br>1.88 GPa	295 K<br>2.40 GPa	295 K<br>3.18 GPa	295 K<br>3.62 GPa
I1···I1^{i}	4.0008(3)	3.9770(3)	3.9564(3)	3.7574(13)	3.7072(18)	3.639(2)	3.6061(15)
C1–I1···I1^{i}	154.22(6)	154.63(5)	154.93(5)	154.62(15)	154.9(3)	155.5(3)	155.5(2)
I1···I1^{i}–C1^{i}	154.22(6)	154.63(5)	154.93(5)	154.62(15)	154.9(3)	155.5(3)	155.5(2)
C1–I1···I1^{i}–C1^{i}	180.00	180.00	180.00	180.00	180.0	180.0	180.0
I1···H11^{ii}	3.39	3.38	3.37	3.17	3.16	3.08	3.06
C1–I1···H11^{ii}	138	138	137	134	134	133	132
I1···H11^{ii}–C1^{ii}	138	138	138	138	138	138	138
C1–I1···H11^{ii}–C1^{ii}	–60	–61	–61	–66	–68	–69	–69
H11···I1^{iii}	3.39	3.38	3.37	3.17	3.16	3.08	3.06
C1–H11···I1^{iii}	138	138	138	138	138	138	138
H11···I1^{iii}–C1^{iii}	138	138	137	134	134	133	132
C1–H11···I1^{iii}–C1^{iii}	–60	–61	–61	–66	–68	–69	–69
I1···H12^{v}	3.39	3.37	3.35	3.13	3.09	3.03	2.99
C1–I1···H12^{v}	87	87	87	86	86	86	86
I1···H12^{v}–C1^{v}	142	142	142	142	141	141	141
C1–I1···H12^{v}–C1^{v}	–99	–99	–99	–96	–95	–97	–96
H12···I1^{vi}	3.39	3.37	3.35	3.13	3.09	3.03	2.99
C1–H12···I1^{vi}	142	142	142	142	141	141	141
H12···I1^{vi}–C1^{vi}	87	87	87	86	86	86	86
C1–H12···I1^{vi}–C1^{vi}	99	99	99	96	95	97	96
I1···H21^{vii}	3.55	3.54	3.53	3.25	3.19	3.15	3.09
C1–I1···H21^{vii}	74	74	74	75	75	75	75
I1···H21^{vii}–C2^{vii}	153	152	152	153	154	149	155
C1–I1···H21^{vii}–C2^{vii}	–9	–8	–7	–11	–17	2	–11
H21···I1^{viii}	3.55	3.54	3.53	3.25	3.19	3.15	3.09
C2–H21···I1^{viii}	153	152	152	153	154	149	155
H21···I1^{viii}–C1^{viii}	74	74	74	75	75	75	75
C2–H21···I1^{viii}–C1^{viii}	–9	–8	–7	–11	–17	2	–11
I1···H22^{ix}	3.42	3.40	3.39	3.19	3.15	3.13	3.08
C1–I1···H22^{ix}	108	108	108	111	112	110	112
I1···H22^{ix}–C2^{ix}	140	140	140	141	142	137	140
C1–I1···H22^{ix}–C2^{ix}	98	99	99	94	89	104	93
H22···I1^{ix}	3.42	3.40	3.39	3.19	3.15	3.13	3.08
C2–H22···I1^{ix}	140	140	140	141	142	137	140
H22···I1^{ix}–C1^{ix}	108	108	108	111	112	110	112
C2–H22···I1^{ix}–C1^{ix}	–98	–99	–99	–94	–89	–104	–93
I1···H22^{x}	3.57	3.56	3.54	3.32	3.26	3.20	3.17
C1–I1···H22^{x}	81	81	80	77	75	78	75
I1···H22^{x}–C2^{x}	120	119	119	117	117	116	116
C1–I1···H22^{x}–C2^{x}	92	92	92	96	100	89	97
H22···I1^{xi}	3.57	3.56	3.54	3.32	3.26	3.20	3.17
C2–H22···I1^{xi}	120	119	119	117	117	116	116
H22···I1^{xi}–C1^{xi}	81	81	80	77	75	78	75
C2–H22···I1^{xi}–C1^{xi}	–92	–92	–92	–96	–100	–89	–97
I1···H23^{xii}	3.31	3.30	3.29	3.09	3.06	2.99	2.98
C1–I1···H23^{xii}	139	139	138	139	138	140	138
I1···H23^{xii}–C2^{xii}	164	164	164	160	157	165	159
C1–I1···H23^{xii}–C2^{xii}	157	157	157	156	159	139	157
H23···I1^{xiii}	3.31	3.30	3.29	3.09	3.06	2.99	2.98
C2–H23···I1^{xiii}	164	164	164	160	157	165	159
H23···I1^{xiii}–C1^{xiii}	139	139	138	139	138	140	138
C2–H23···I1^{xiii}–C1^{xiii}	157	157	157	156	159	139	157

Symmetry codes: (i) 1 – *x* , 2 – *y*, 2 – *z*; (ii) 1 + *x*, *y*, *z*; (iii) –1 + *x*, *y*, *z*; (v) 1/2 + *x*, 3/2 – *y*, 1/2 + *z*; (vi) –1/2 + *x*, 3/2 – *y*, –1/2 + *z*; (vii) –1/2 – *x*, 1/2 + *y*, 3/2 – *z*; (viii) –1/2 – *x*, –1/2 + *y*, 3/2 – *z*; (ix) –*x*, 2 – *y*, 2 – *z*; (x) 1/2 + *x*, 3/2 – *y*, –1/2 + *z*; (xi) –1/2 + *x*, 3/2 – *y*, 1/2 + *z*; (xii) 1/2 – *x*, 1/2 + *y*, 3/2 – *z*; (xiii) 1/2 – *x*, –1/2 + *y*, 3/2 – *z*.

Table S13. Hydrogen bonds (potential) geometries (Å, °) for **MBE** at variable temperature and pressure conditions, compared to those commensurate with the sums of the van der Waals radii of Br and H atoms at 295 K/3.87 GPa (Bondi, 1964).

	D–H	H···A	D···A	D–H···A
temperature/pressure			140 K/0.1 MPa	
C1–H12···Br1^{iv}	0.97	3.45	4.048(3)	122
C1–H12···Br1^{vi}	0.97	3.37	4.137(3)	138
C2–H21···Br1^{iii}	0.96	3.47	3.932(3)	112
C2–H22···Br1^{ix}	0.96	3.30	4.069(3)	138
C2–H22···Br1^{xi}	0.96	3.42	4.005(2)	122
C2–H23···Br1^{xiii}	0.96	3.14	4.062(3)	163
temperature/pressure			120 K/0.1 MPa	
C1–H12···Br1^{iv}	0.97	3.45	4.041(3)	121
C1–H12···Br1^{vi}	0.97	3.33	4.105(3)	138
C2–H21···Br1^{iii}	0.96	3.48	3.924(3)	110
C2–H22···Br1^{ix}	0.96	3.27	4.043(3)	139
C2–H22···Br1^{xi}	0.96	3.40	3.980(3)	121
C2–H23···Br1^{xiii}	0.96	3.14	4.056(3)	160
temperature/pressure			100 K/0.1 MPa	
C1–H12···Br1^{iv}	0.97	3.45	4.036(3)	121
C1–H12···Br1^{vi}	0.97	3.31	4.084(3)	138
C2–H21···Br1^{iii}	0.96	3.48	3.914(3)	110
C2–H22···Br1^{ix}	0.96	3.25	4.022(3)	138
C2–H22···Br1^{xi}	0.96	3.38	3.962(2)	121
C2–H23···Br1^{xiii}	0.96	3.14	4.052(3)	160
temperature/pressure			295 K/1.83 GPa	
C1–H12···Br1^{iv}	0.97	3.24	3.789(4)	118
C1–H12···Br1^{vi}	0.97	3.11	3.876(4)	137
C2–H21···Br1^{iii}	0.96	3.42	3.732(4)	101
C2–H22···Br1^{ix}	0.96	3.06	3.852(4)	141
C2–H22···Br1^{xi}	0.96	3.20	3.750(3)	118
C2–H23···Br1^{xiii}	0.96	2.99	3.857(4)	151
temperature/pressure			295 K/2.13 GPa	
C1–H12···Br1^{iv}	0.97	3.22	3.766(4)	117
C1–H12···Br1^{vi}	0.97	3.08	3.841(4)	137
C2–H21···Br1^{iii}	0.96	3.35	3.706(4)	104
C2–H22···Br1^{ix}	0.96	3.04	3.820(4)	140
C2–H22···Br1^{xi}	0.96	3.17	3.727(3)	118
C2–H23···Br1^{xiii}	0.96	2.95	3.837(3)	155
temperature/pressure			295 K/3.07 GPa	
C1–H12···Br1^{iv}	0.97	3.13	3.674(9)	117
C1–H12···Br1^{vi}	0.97	3.01	3.758(9)	135
C2–H21···Br1^{iii}	0.96	3.22	3.651(9)	109
C2–H22···Br1^{ix}	0.96	3.02	3.764(10)	135
C2–H22···Br1^{xi}	0.96	3.07	3.635(9)	119
C2–H23···Br1^{xiii}	0.96	2.84	3.771(9)	162
temperature/pressure			295 K/3.87 GPa	
C1–H12···Br1^{iv}	0.97	3.04	3.562(19)	115
C1–H12···Br1^{vi}	0.97	2.97	3.709(19)	134
C2–H21···Br1^{iii}	0.96	2.91	3.560(19)	126
C2–H22···Br1^{ix}	0.96	3.05	3.67(2)	124
C2–H22···Br1^{xi}	0.96	3.04	3.573(17)	116
C2–H23···Br1^{xiii}	0.96	2.83	3.77(2)	165

Symmetry codes: (iii) –1 + *x*, *y*, *z*; (iv) –*x*, 2 – *y*, 1 – *z*; (vi) –1/2 + *x*, 3/2 – *y*, –1/2 + *z*; (ix) –*x*, 2 – *y*, 2 – *z*; (xi) –1/2 + *x*, 3/2 – *y*, 1/2 + *z*; (xiii) 1/2 – *x*, –1/2 + *y*, 3/2 – *z*.

Table S14. Hydrogen bonds (potential) geometries (Å, °) for **MIE** at variable temperature and pressure conditions, compared to those commensurate with the sums of the van der Waals radii of I and H atoms at 295 K/3.62 GPa (Bondi, 1964).

	D–H	H···A	D···A	D–H···A
temperature/pressure			140 K/0.1 MPa	
C1–H11···I1^{iii}	0.97	3.39	4.167(2)	138
C1–H12···I1^{vi}	0.97	3.39	4.198(2)	142
C2–H21···I1^{viii}	0.96	3.55	4.4224(19)	153
C2–H22···I1^{ix}	0.96	3.42	4.199(2)	140
C2–H22···I1^{xi}	0.96	3.57	4.133(2)	120
C2–H23···I1^{xiii}	0.96	3.31	4.236(2)	164
temperature/pressure			120 K/0.1 MPa	
C1–H11···I1^{iii}	0.97	3.38	4.155(2)	138
C1–H12···I1^{vi}	0.97	3.37	4.177(2)	142
C2–H21···I1^{viii}	0.96	3.54	4.4094(18)	152
C2–H22···I1^{ix}	0.96	3.40	4.184(2)	140
C2–H22···I1^{xi}	0.96	3.56	4.1147(19)	119
C2–H23···I1^{xiii}	0.96	3.30	4.2290(19)	164
temperature/pressure			100 K/0.1 MPa	
C1–H11···I1^{iii}	0.97	3.37	4.147(2)	138
C1–H12···I1^{vi}	0.97	3.35	4.160(2)	142
C2–H21···I1^{viii}	0.96	3.53	4.3988(18)	152
C2–H22···I1^{ix}	0.96	3.39	4.1681(19)	140
C2–H22···I1^{xi}	0.96	3.54	4.0967(18)	119
C2–H23···I1^{xiii}	0.96	3.29	4.2232(19)	164
temperature/pressure			295 K/1.88 GPa	
C1–H11···I1^{iii}	0.97	3.17	3.942(12)	138
C1–H12···I1^{vi}	0.97	3.13	3.939(6)	142
C2–H21···I1^{viii}	0.96	3.25	4.128(10)	153
C2–H22···I1^{ix}	0.96	3.19	3.983(6)	142
C2–H22···I1^{xi}	0.96	3.32	3.857(6)	117
C2–H23···I1^{xiii}	0.96	3.09	4.009(9)	160
temperature/pressure			295 K/2.40 GPa	
C1–H11···I1^{iii}	0.97	3.16	3.928(16)	138
C1–H12···I1^{vi}	0.97	3.09	3.898(10)	141
C2–H21···I1^{viii}	0.96	3.19	4.073(14)	154
C2–H22···I1^{ix}	0.96	3.15	3.949(10)	142
C2–H22···I1^{xi}	0.96	3.26	3.803(10)	117
C2–H23···I1^{xiii}	0.96	3.06	3.964(14)	157
temperature/pressure			295 K/3.18 GPa	
C1–H11···I1^{iii}	0.97	3.08	3.853(14)	138
C1–H12···I1^{vi}	0.97	3.03	3.833(12)	141
C2–H21···I1^{viii}	0.96	3.15	4.004(14)	149
C2–H22···I1^{ix}	0.96	3.13	3.894(10)	137
C2–H22···I1^{xi}	0.96	3.20	3.731(11)	116
C2–H23···I1^{xiii}	0.96	2.99	3.924(13)	165
temperature/pressure			295 K/3.62 GPa	
C1–H11···I1^{iii}	0.97	3.06	3.837(14)	138
C1–H12···I1^{vi}	0.97	2.99	3.795(8)	141
C2–H21···I1^{viii}	0.96	3.09	3.974(11)	155
C2–H22···I1^{ix}	0.96	3.08	3.864(8)	140
C2–H22···I1^{xi}	0.96	3.17	3.693(7)	116
C2–H23···I1^{xiii}	0.96	2.98	3.886(10)	159

Symmetry codes: (iii) –1 + *x*, *y*, *z*; (vi) –1/2 + *x*, 3/2 – *y*, –1/2 + *z*; (viii) –1/2 – *x*, –1/2 + *y*, 3/2 – *z*; (ix) –*x*, 2 – *y*, 2 – *z*; (xi) –1/2 + *x*, 3/2 – *y*, 1/2 + *z*; (xiii) 1/2 – *x*, –1/2 + *y*, 3/2 – *z*.

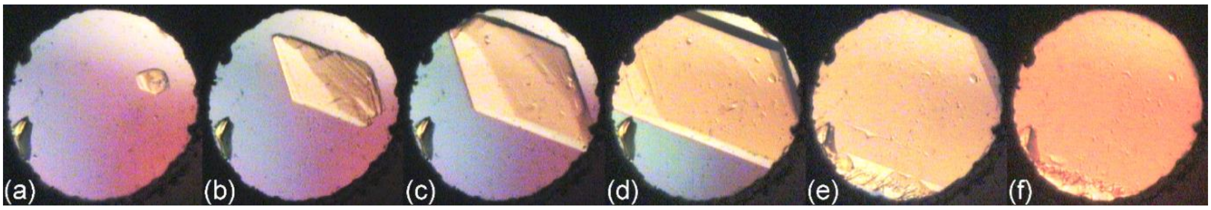

Figure S1. Stages of isochoric, with decreasing temperature, growth of the **MBE** single crystal in a diamond-anvil cell initially pressurized, at ambient temperature to *ca.* 2 GPa, started at 350(3) K (a), leading to the sample fully filling the high-pressure chamber at 1.83 GPa (f). The pressure chamber is 0.37 mm in diameter. The ruby chip, for pressure calibration, is located at the down-left side of the high-pressure chamber.

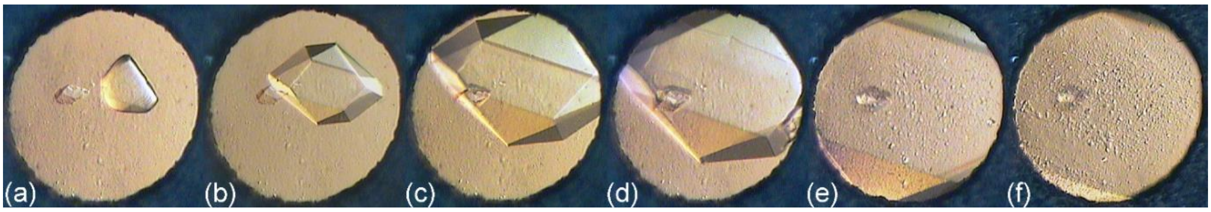

Figure S2. Stages of isochoric, with decreasing temperature, growth of the **MIE** single crystal in a diamond-anvil cell initially pressurized, at ambient temperature to *ca.* 2 GPa, started at 365(3) K (a), leading to the sample fully filling the high-pressure chamber at 1.88 GPa (f). The pressure chamber is 0.37 mm in diameter. The ruby chip, for pressure calibration, is located at the middle-left side of the high-pressure chamber.

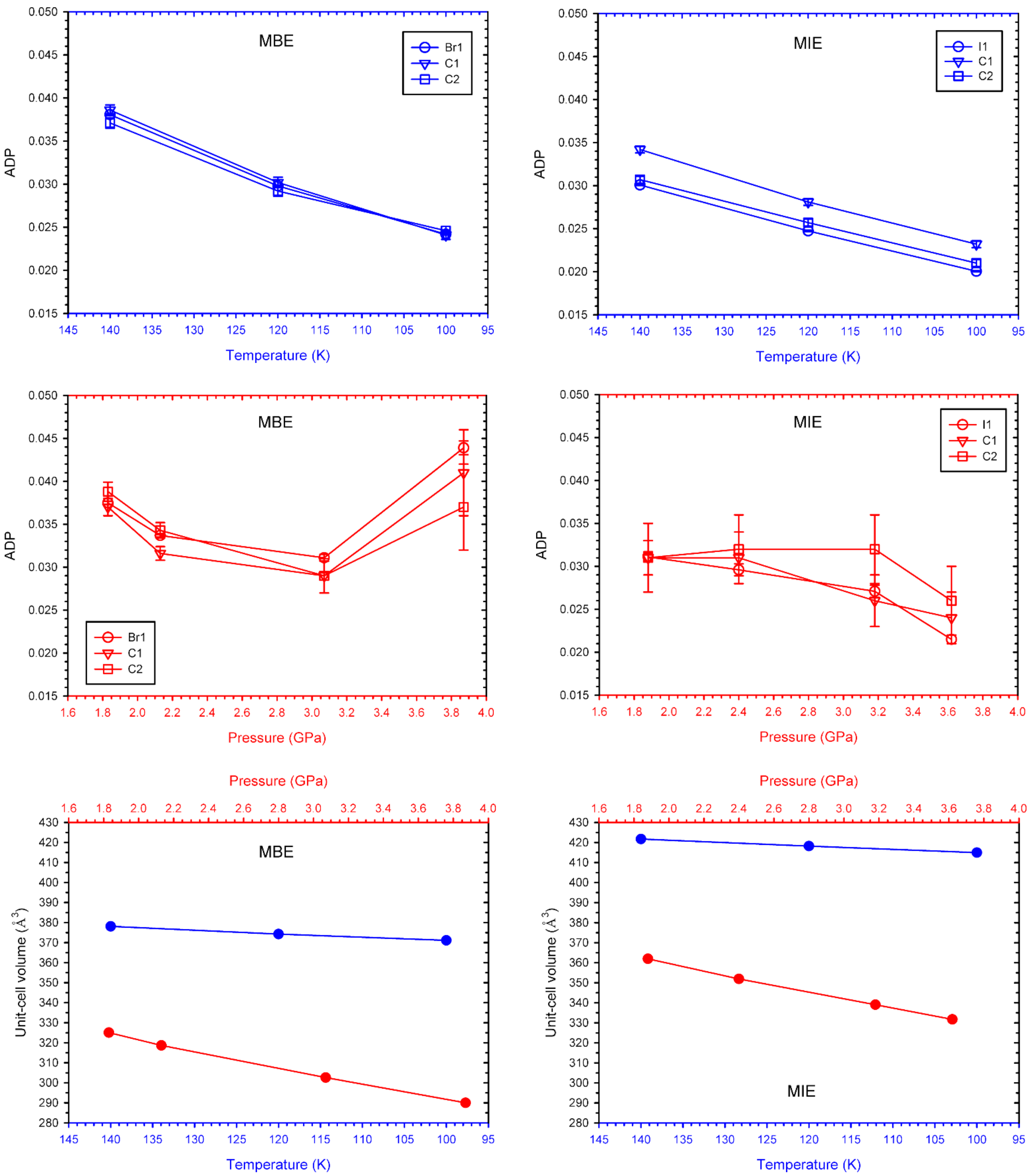

Figure S3. Comparison of the average ADP values for C1, C2 and X (X = Br for **MBE** and X = I for **MIE**) atoms as well as unit-volume changes at variable investigated temperature and pressure points. The error bars for ADP, at different pressure points, are indicated. The lines joining the points are guides for the eye only.

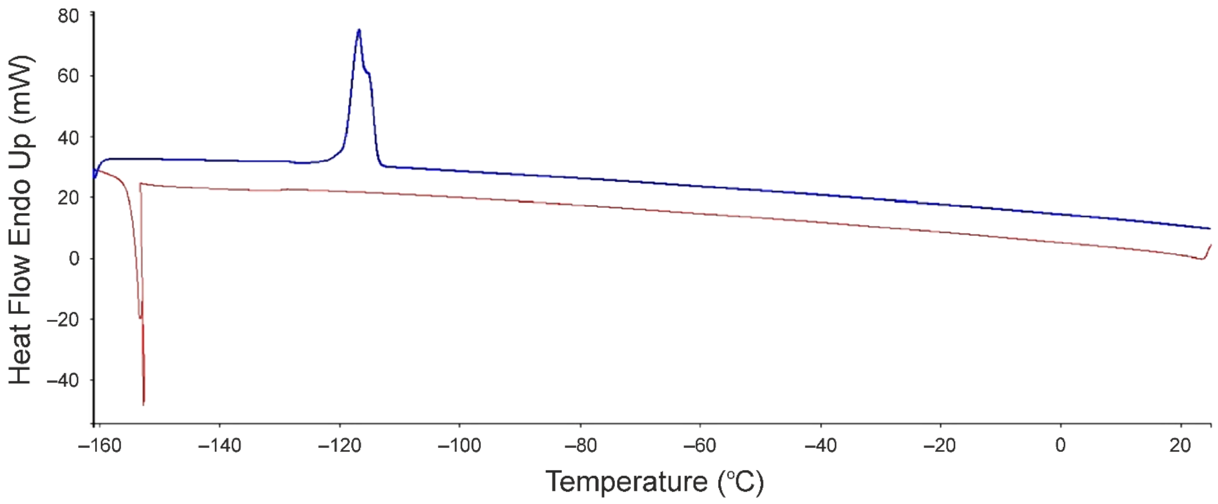

Figure S4. DSC cooling and heating runs measured at the rate of 10 K·min^{-1}, at ambient pressure, for **MBE**.

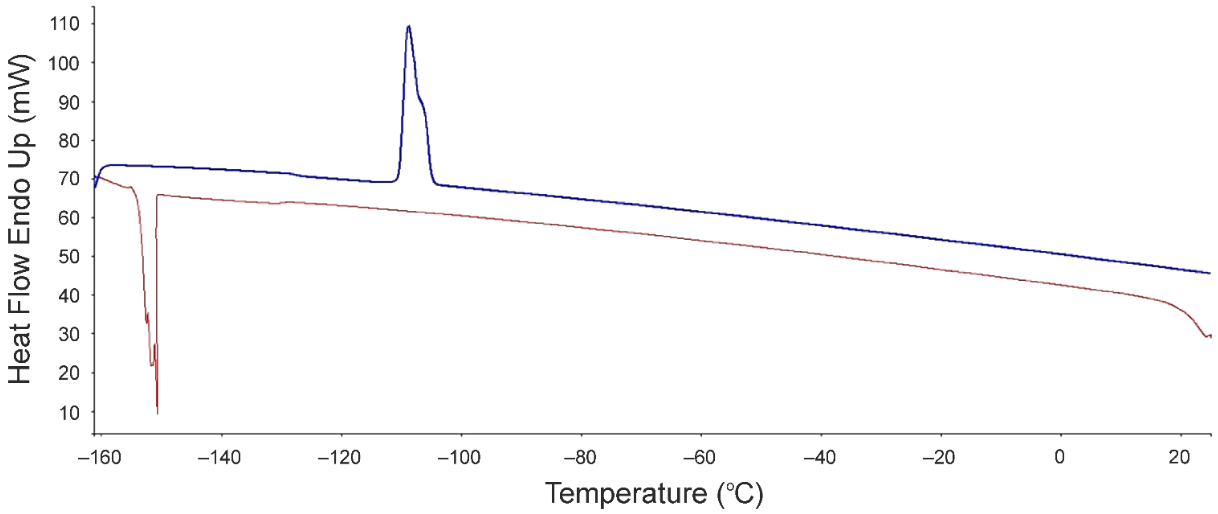

Figure S5. DSC cooling and heating runs measured at the rate of 10 K·min^{-1}, at ambient pressure, for **MIE**.

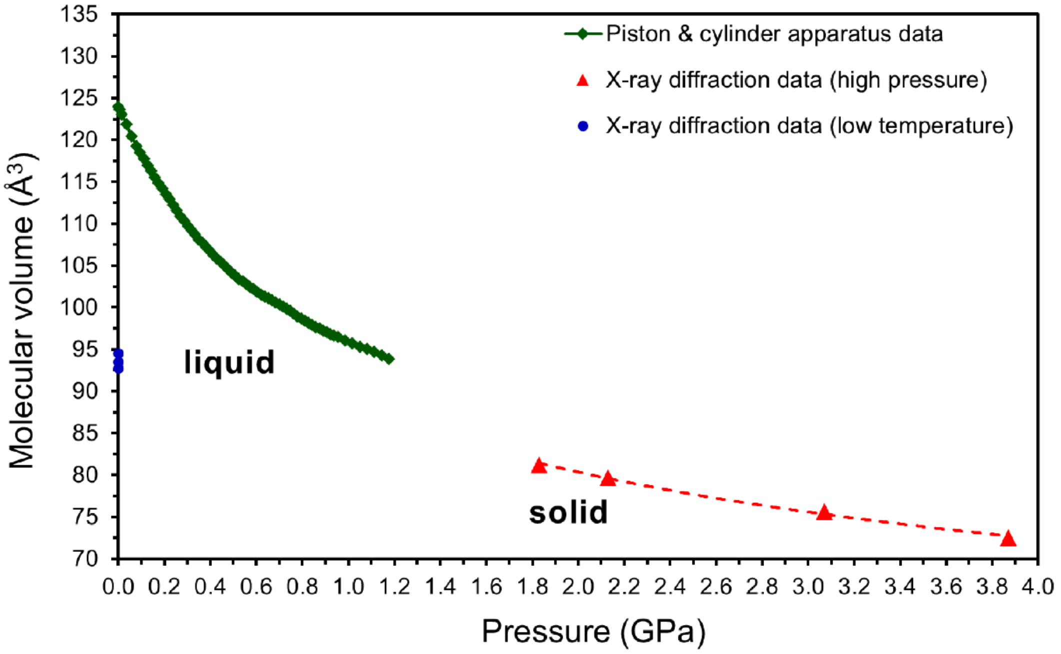

Figure S6. Molecular volume of **MBE** at room temperature as a function of pressure measured in the piston-and-cylinder press (green diamonds). The volumes measured at high pressure (red triangles) and low temperature (blue circles) by single-crystal X-ray diffraction are indicated. The red dashed line is for guiding the eye only.

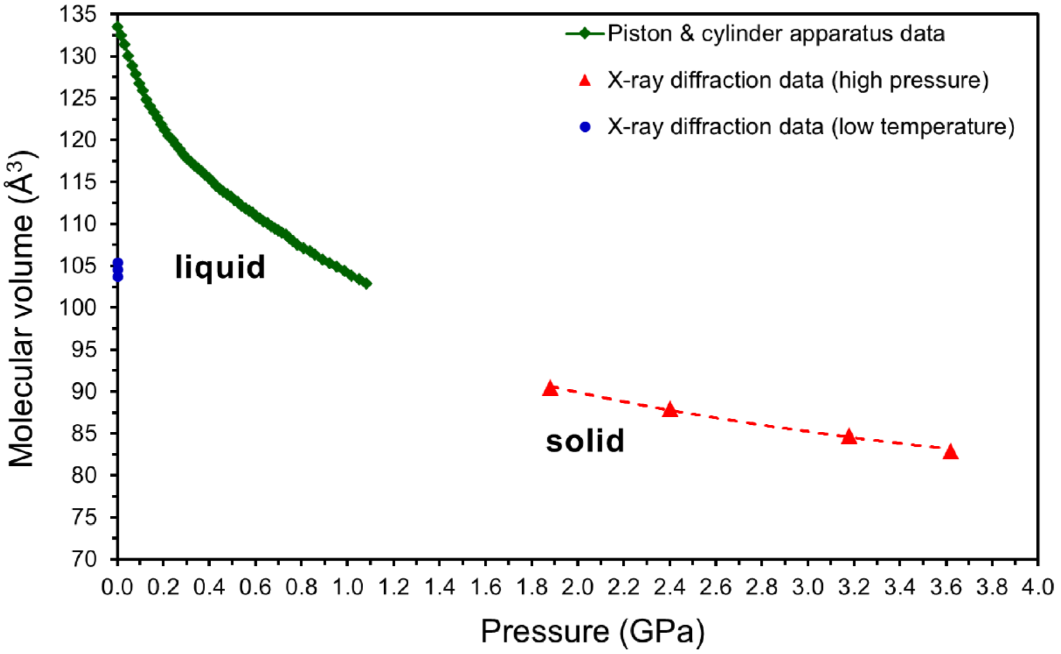

Figure S7. Molecular volume of **MIE** at room temperature as a function of pressure measured in the piston-and-cylinder press (green diamonds). The volumes measured at high pressure (red triangles) and low temperature (blue circles) by single-crystal X-ray diffraction are indicated. The red dashed line is for guiding the eye only.

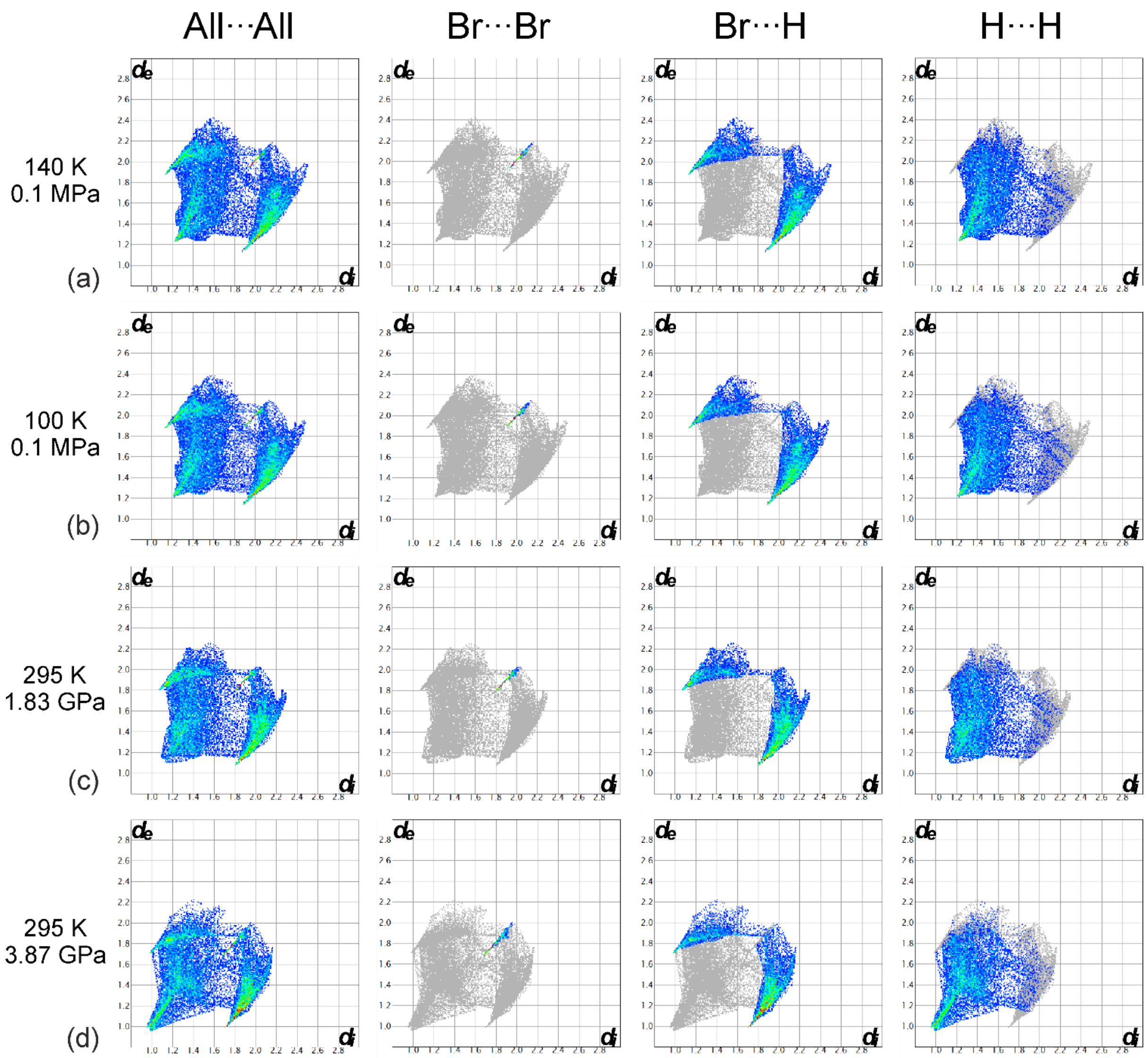

Figure S8. Two-dimensional fingerprint plots of **MBE** determined at: 140 K/0.1 MPa (a); 100 K/0.1 MPa (b); 295 K/1.83 GPa (c) and 295 K/3.87 GPa (d). The Br···Br, Br···H and H···H contacts are highlighted.

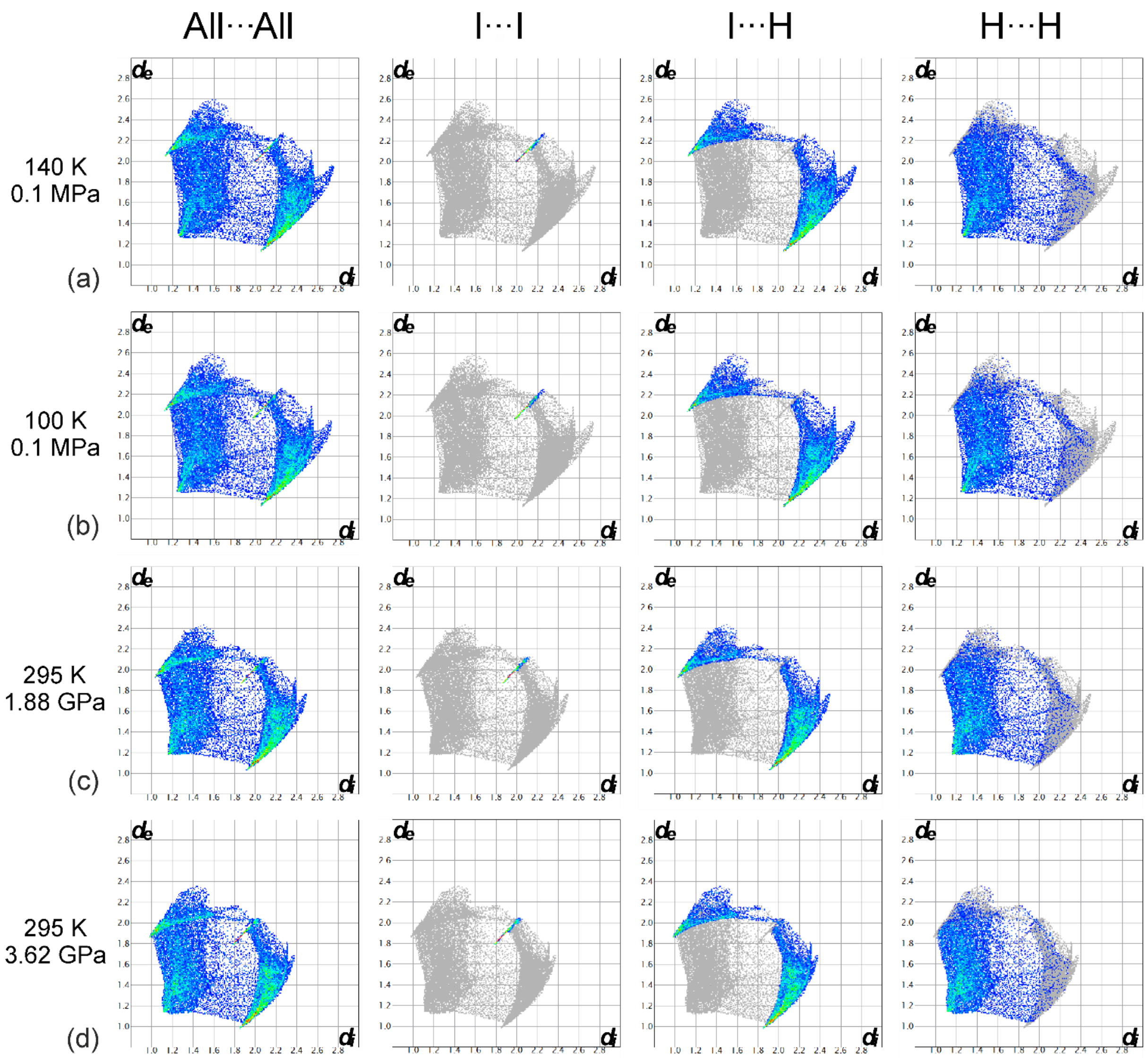

Figure S9. Two-dimensional fingerprint plots of **MIE** determined at: 140 K/0.1 MPa (a); 100 K/0.1 MPa (b); 295 K/1.88 GPa (c) and 295 K/3.62 GPa (d). The I···I, I···H and H···H contacts are highlighted.

References (SI)

Bondi, A. (1964). *J. Phys. Chem.* **68**, 441–451.

Cliffe, M. J. & Goodwin, A. L. (2012). *J. Appl. Cryst.* **45**, 1321–1329.
